# Redirecting Excited‐State Proton Transfer Through Supramolecular Polymerization in Nanoconfinement

**DOI:** 10.1002/anie.7594145

**Published:** 2026-06-08

**Authors:** Luis C. Pantaleone, Robert Hutchings, Bente Reus, Jacopo Martinelli, Alessia Lasorsa, Patrick C. A. van der Wel, Marc C. A. Stuart, Wesley R. Browne, Tibor Kudernac

**Affiliations:** ^1^ Stratingh Institute for Chemistry University of Groningen Groningen the Netherlands; ^2^ Zernike Institute for Advanced Materials University of Groningen Groningen the Netherlands

**Keywords:** nano‐confinement, photoacids, proton transfer, supramolecular polymerization

## Abstract

The photoluminescence of photoacids in supramolecular assemblies provides crucial insights into proton transfer (PT) processes within biologically relevant confinement. In this work, we present a strategy to activate intermolecular excited‐state PT within the hydrophobic cavities of cyclodextrin‐based nanotubes. Activation is achieved using a specifically designed amphoteric emitter which undergoes a pKa inversion in the photoexcited state. Despite this photophysical behavior, intramolecular PT does not occur due to the spatial separation between the proton donor and acceptor sites in the compound. However, in the presence of γ‐cyclodextrin, the photoacid assembles into guest pairs, enabling pre‐organized PT between neighboring molecules. The effects of confinement on photostability, emission lifetime, and quantum yield indicate a mechanistic shift from excited‐state protolytic dissociation to intermolecular excited‐state PT. Spectroscopic investigation of the assembly mechanism and solvent isotope effect further supports the role of a template effect, reminiscent of enzymatic activation, in facilitating PT in the excited state.

## Graphical abstract

Supramolecular confinement redirects the excited‐state proton transfer pathway of an amphoteric emitter. In water, the molecule undergoes ESPD, whereas co‐assembly with γ‐cyclodextrin forms nanotubes that template slip‐stacked emitter pairs and enable ESIMPT. Structural and photophysical characterization of the assemblies shows that the preorganization of emitters induced by the cyclodextrin cavity, not simple self‐aggregation, drives this mechanistic transition.

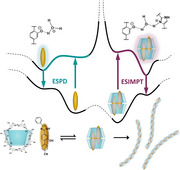

## 1 Introduction

Proton transfer (PT) is a critical process in biochemical transformations, playing a key role in diverse contexts such as bio‐catalysis, transport channels, and genetic replication [1, 2]. An approach to investigate these processes in biologically relevant confinements is using spectroscopic probes based on excited‐state proton transfer (ESPT) [3]. ESPT can occur either intramolecularly (ESIPT), typically leading to tautomer or isomer formation, or bimolecularly, as seen in acids that undergo a significant increase in dissociation constant upon electronic excitation, resulting in protolytic dissociation into surrounding water (ESPD) [4]. The photoluminescence of these probes offers a fast and sensitive physical method to observe the influence of the microenvironment on PT and shows that the effects of confinement largely depend upon the molecularity of these mechanisms. ESIPT, which does not depend upon an external proton donor or acceptor, was reported in the cavities of inclusion complexes, micelles, and nanotubes, where it benefits from the absence of competing structured hydrogen bond networks and even in polar aprotic solvents and soft condensed matter [5, 6, 7, 8]. In contrast, ESPD of photoacids is deactivated under these conditions, as interfacial water in hydrophobic confinements rarely participates in PT, and when it does, the viscosity of these water nanopools promotes the geminal recombination in the excited state [9, 10, 11]. A few approaches to promote bimolecular ESPT have already been explored, for example, transferring the proton to an external base or through the self‐aggregation of amphoteric compounds [12, 13, 14, 15]. However, the activation of ESPT via template effect has received minimal attention, despite the widespread role of structured microenvironments in directing PT in biological systems—such as protonation of proximal residues in protein binding pockets or multistep proton conduction between DNA base pairs [16, 17, 18].

**SCHEME 1:**
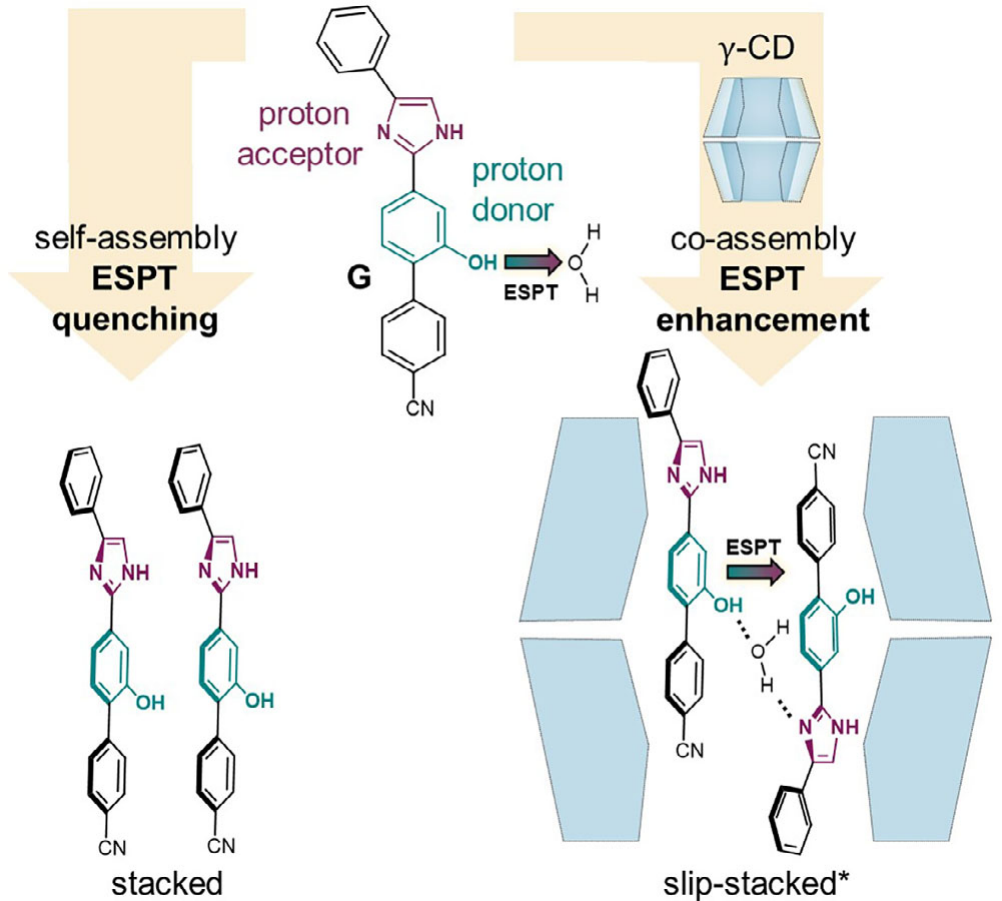
Co‐assembling the emitter with γ‐CD changes the ESPT mechanism from ESPD to intermolecular ESPT. *Both parallel and antiparallel transition dipole moment arrangements are possible in slip‐stacked configuration.

Pre‐organizing proton donor and acceptor sites within hydrophobic cavities offers a promising strategy to activate intermolecular ESPT (ESIMPT) mechanisms in supramolecular confinement. With this concept in mind, we designed an amphoteric emitter (G) with photoacidic properties. When co‐assembled with γ‐cyclodextrin (γ‐CD), the photoacid undergoes a shift in its ESPT mechanism due to the pre‐organization of its proton donor and acceptor sites within the cavities of supramolecular nanotubes (Scheme 1).

Beyond its biological relevance, studying how non‐covalent interactions within supramolecular assemblies influence (photo)chemical pathways may expand the currently limited catalytic applications of ESPT photoacids [19, 20, 21, 22, 23, 24, 25]. If effective in directing ESPT on a proton‐responsive substrate, this approach—using a tailored structured microenvironment to pre‐organize the photoacid and substrate—could help overcome a key challenge in ESPT catalysis by promoting PT to reactive sites while minimizing competition from H‐bonding solvents within the excited‐state lifetime [26].

## 2 Results and Discussion

### 2.1 Photoacidic Behavior of the Amphoteric Emitter

**SCHEME 2:**
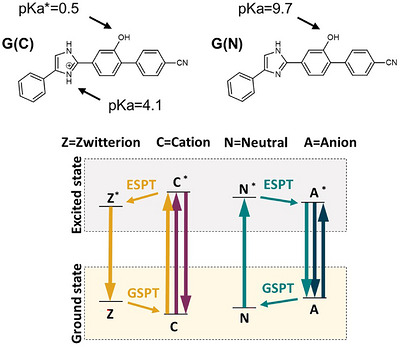
The photophysical behavior of G in water.

**FIGURE 1:**
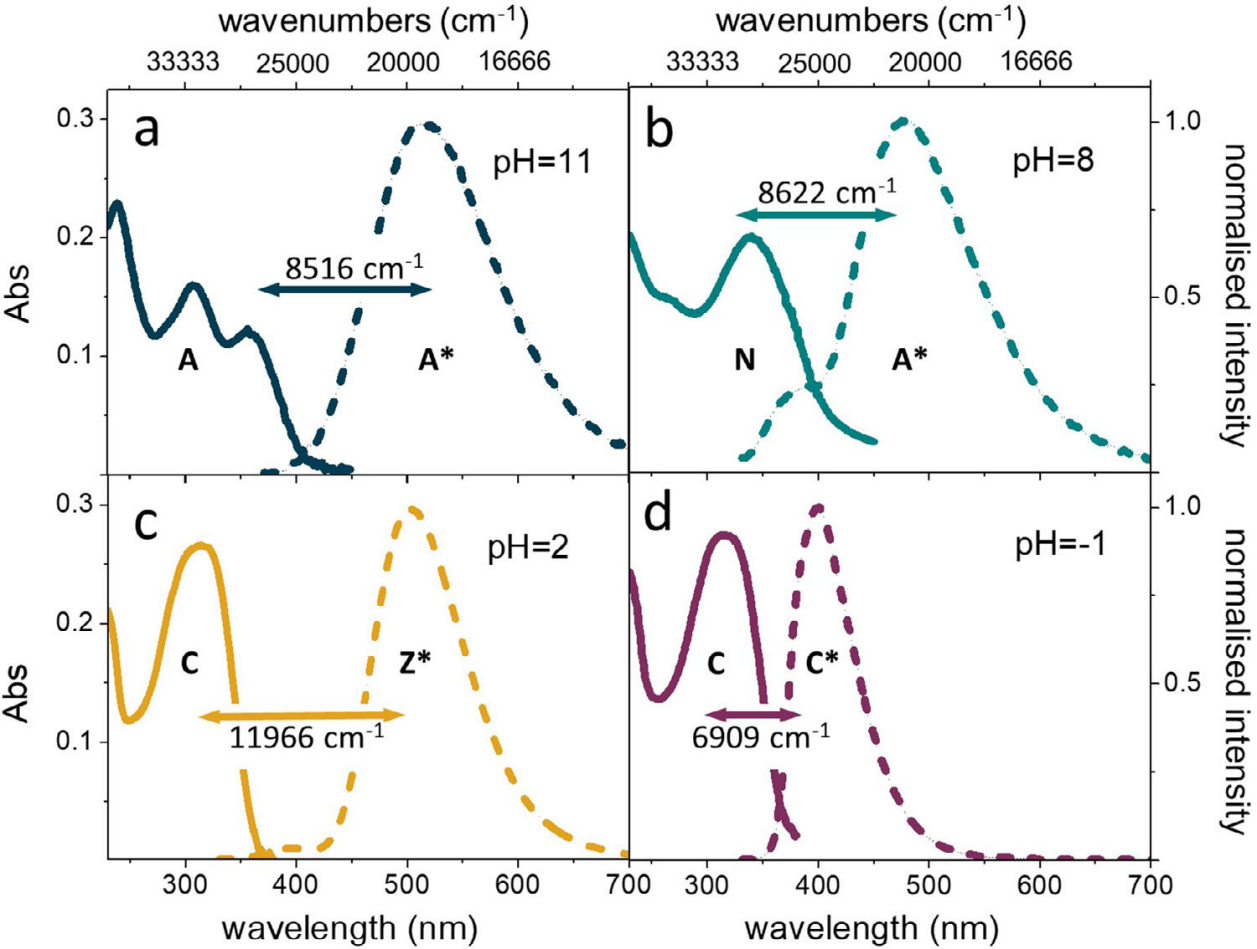
Steady state spectroscopy. (a) (b) (c) (d) Absorption and emission spectra of G as a function of pH.

The photophysical behavior of G in water and its pKa in the ground and excited states were determined by spectroscopic titrations. The pH dependence of the UV/vis absorption of G reveals two distinct acid/base equilibria, corresponding to those of the imidazole (pKa = 4.1 ± 0.1) and phenol (9.7 ± 0.2) moieties (Scheme 2). These transitions were respectively identified by a hypsochromic shift upon formation of the imidazolium cation G(C) (*λ*_max_ 314 nm), and by the coalescence of the absorption bands at 309 and 358 nm upon protonation of the phenolate anion G(A) (Figures 1a–c and S1a). The appearance of the neutral form G(N), which absorbs with λ_max_ 338 nm, resulted, in both titrations, in an increase in light scattering due to the low solubility of this species. The presence of γ‐CD had little influence on the acid/base chemistry (pKa_1_ = 4.4 ± 0.3, pKa_2_ = 9.7 ± 0.2); however, the neutral form of the G was more soluble, and the absorptions of both G(N) and G(C) were red‐shifted (Figure S1b).

The pH dependence of fluorescence emission reflects the acid‐base chemistry of the excited state (Scheme 2). Comparison of the emission at pH 8 and pH 11 reveals a similar Stokes shift and no appreciable difference in emission lifetime upon photoexcitation of G(N) and G(A) forms (Figure 1a,b). Both emissions originate from the radiative relaxation of the same species G(A)*, indicating a PT in the excited state that follows the photoexcitation of G(N). Upon protonation of the imidazole ring in the ground state, the quantum yield of emission increases significantly (ϕG(Z*)/ϕG(A*) = 5.0) (Figures 1b,c and S11a,b). The positive charge developed on the cation increases the acidity of the phenol group in the excited state, ESPT is promoted, and the emission occurs from the stabilized zwitterion G(Z)* (Scheme 2). Titration of the phenolic proton in the excited state was observed around pKa* ∼ 0.5 (Figure S1c). In this pH range, the band corresponding to the emission from the zwitterion was suppressed, and a new emission was observed, characterized by a reduced Stokes shift. This new emission was assigned to the relaxation from the cationic form of G(C)* (Scheme 2) (Figure 1c,d). The latter spectral transition occurred without changes in the electronic absorption.

Besides confirming the photoacidic properties of G, these experimental results show a pKa inversion between the ground and excited states. The fact that G(N)* emission was never detected by steady state spectroscopy means that during the titrations, the protonation of the imidazole site in the ground state occurs prior to the titration of the phenolic proton in the excited state. Thus, the phenol is less acidic than the imidazolium ion in the ground state, but this relationship reverses upon photoexcitation.

Despite the pKa inversion, the separation of the proton donor and acceptor sites in the molecular design prevents ESIPT. The absence of an intramolecular hydrogen bond is consistent with the solvatochromic behavior of G in dry dichloromethane: the Stokes shift decreases (*λ* ^−1^ = 6864 cm^−1^) due to a hypsochromic change in emission, resembling the spectral shift observed during the formation of G(C*) band in concentrated hydrochloric acid (Figures 1d and S2) [7, 13]. Thus, the photoluminescence in polar aprotic solvents likely occurs via an ICT mechanism rather than ESIPT.

The possibility of a proton wire connecting donor and acceptor sites, reported in a few amphoteric emitters [27, 28, 29], was ruled out by the negligible effect of chaotropic salt on the emission intensity (Figure S3). This indicates that the photoluminescence of G(N) in water can only proceed via the ESPD mechanism.

Determination of the phenol's ΔpKa between G(N) and G(N)* using direct titration or the Förster cycle was not possible, since both methods require the spectroscopic observation of the transient species G(N)* [30]. However, protonation of the imidazole ring in the ground state is expected to enhance the acidity of G(C)* phenol relative to G(N)*. Therefore, using the pKa values of G(C) imidazolium ion and G(C)* phenol as bounds, we estimate that upon photoexcitation of G(N), the phenol undergoes a significant acidity increase, ranging from 5.6 to 9.2 pKa units.

### 2.2 Self‐Aggregation in Water

During the spectroscopic titrations, we observed the presence of a shoulder in the emission spectra (*λ*_max_ = 368 nm) over the pH range dominated by the G(N) species (5 < pH < 9) (Figure 1b). The fact that the ratio between these two bands is not significantly affected in this broad range of acidity excluded an additional acid/base equilibrium. The hypothesis of a double emission from the excitation of G(N), which is possible in some ESPT emitters [31], was also discarded because the photoluminescence of G(N) was found to be dependent on the excitation wavelength (Figure S4). This behavior indicates that G must exist in two distinct forms in the ground state, which can usually be explained by the presence of a conformational equilibrium or by self‐aggregation [31].

**FIGURE 2:**
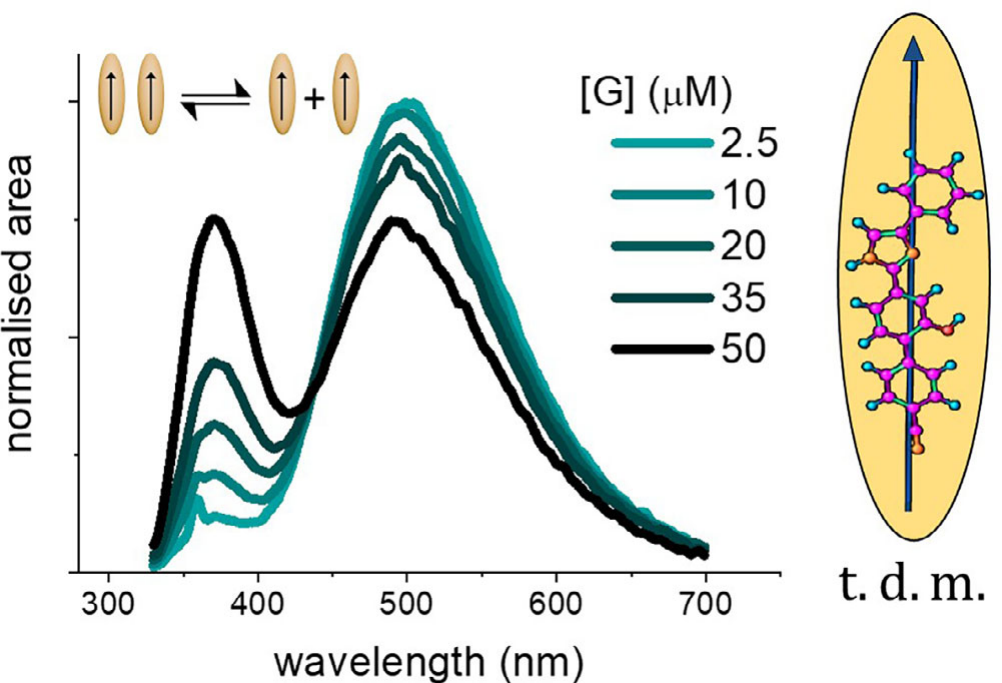
Changes in the emission upon self‐aggregation of G and representation of its transition dipole moment.

The latter option proved to be the case, as evidenced by the concentration dependence of the ratio of intensities of the two bands (Figure 2). The self‐aggregation of G enhances the intensity of the shoulder emission at *λ*_max_ = 368 nm, and, comparing the excitation spectra of the two bands, the absorption of the monomer (*λ*_max_ = 328 nm) is also shifted to shorter wavelengths upon self‐aggregation (*λ*_max_ = 295 nm) (Figures 2 and S4).

Based on the large decrease in the Stokes shift (*λ* ^−1^ = 6724 cm^−1^), the mechanisms of photo‐relaxation of these aggregates clearly differ from ESPT. Considering the hypsochromic shift in absorption, we attributed the aggregation‐induced‐emission band to the photoluminescence of an exciton species (H*). Coulomb coupling between neighboring chromophores can induce such spectral changes when the transition dipole moment (TDM) of π‐conjugated molecules assumes a side‐by‐side—or stacked—assembling configuration [32, 33]. The transition dipole moment of the main electronic absorption band of G was calculated for an accurate structural representation of these aggregates (Figure 2). In this configuration, likely driven by the π‐stacking of the emitters, the proton donor and acceptor sites remain distantly positioned (Scheme 1). This explains why intermolecular ESPT is not activated by the self‐aggregation of G, despite the amphoteric nature of the emitter. This interpretation is also consistent with the monomer's ESPT band dominating the emission spectrum, as H‐type excitons usually exhibit a weak photoluminescence that is forbidden by symmetry rules.

### 2.3 Supramolecular Polymerization of γ‐CD Nanotubes

**FIGURE 3:**
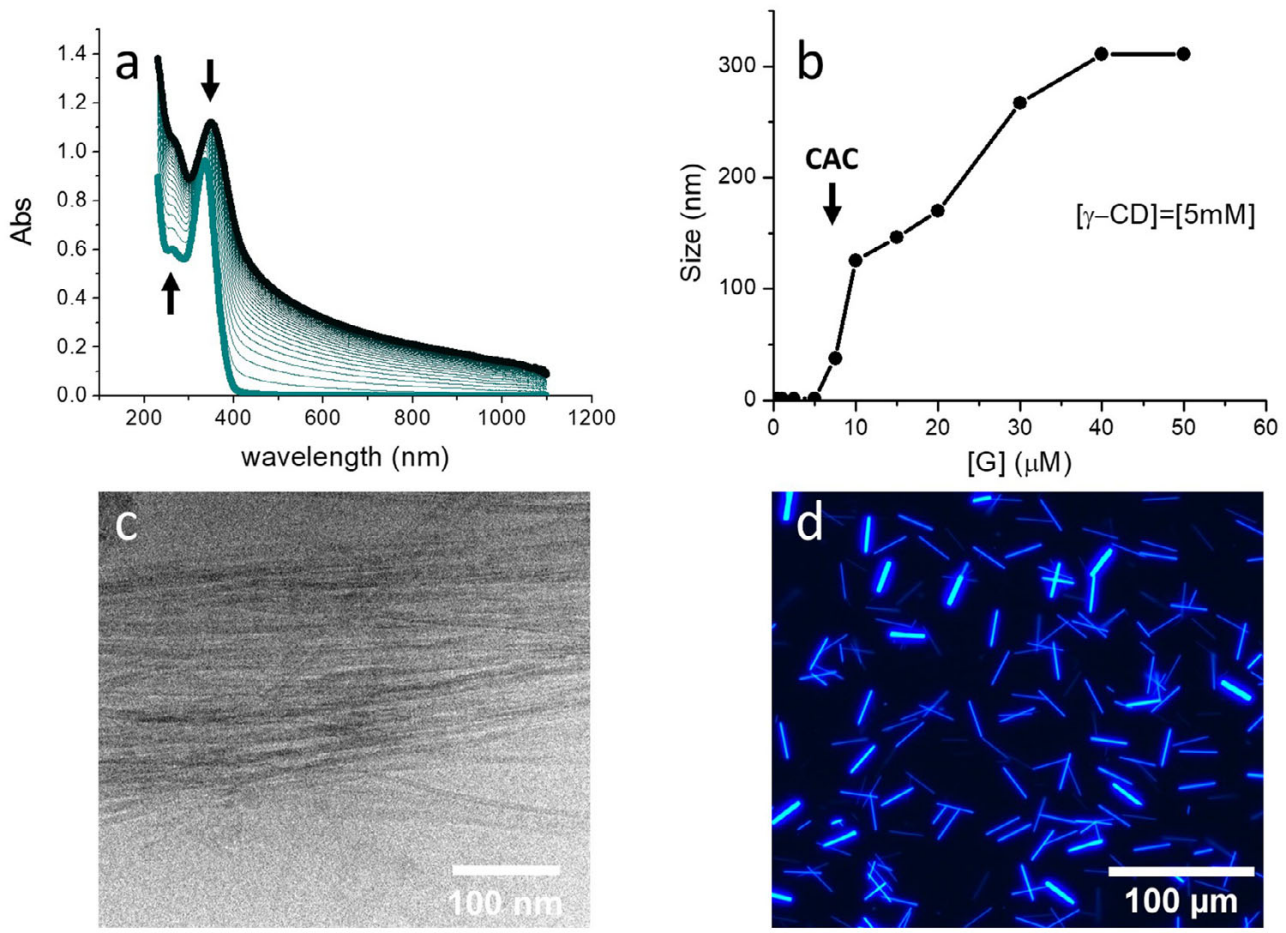
(a) Spectral changes in the absorption during supramolecular polymerization over 30 min. (b) Critical aggregation concentration from DLS data. Plotted sizes represent the mode of the scattering intensity distributions. (c) Cryo‐EM micrograph showing individual nanotubes formed upon co‐assembling G and γ‐CD. (d) Epifluorescence microscopy of rod shaped microcrystals.

In the presence of γ‐CD, the absorption spectrum of G changes (Figure 3a). Within seconds of sugar addition, a rapid spectral transition occurs, marked by the emergence of a shoulder band at *λ* = 280 nm, followed by a slower drift of the main absorption band (Figure 3a). The latter bathochromic shift coincides with an increase in the Tindal scattering.

The two asynchronous spectral changes are attributed to a rapid pre‐equilibrium arising from host‐guest binding between γ‐CD and G, followed by the slower supramolecular polymerization of the resulting inclusion complexes. The red shift of the main absorption band indicates that, while π‐stacking in water results in a side‐by‐side stacked packing, the cyclodextrin cavities favor a slipped‐stacked configuration of the chromophores [32, 33]. Aggregates with such a configuration are usually referred to as J‐type.

Dynamic light scattering (DLS) showed the formation of aggregates with sizes in the range of hundreds of nanometers (Figures 3b and S5a). Upon titrating increasing amounts of G in the presence of excess γ‐CD (5 mM), the inclusion complexes reached their critical aggregation concentration (CAC) at 7.5 µM of G, as indicated by the DLS data (Figures 3b and S5a). Based on the kinetic profile obtained by plotting scattered intensity over time, 90% of the supramolecular polymerization occurs within 15 min of mixing (Figure S5b).

Cyclodextrins are prone to form stacked assemblies since the hydroxyl groups in the outer rims of the macrocycle can hydrogen bond to other sugar units [34]. It has been reported that when the formation of an inclusion complex stabilizes the interaction between macrocycles through guest‐guest hydrogen bonding or π–π interactions, the host‐guest binding can lead to supramolecular polymerization of γ‐CD nanotubes [35]. This structural interpretation was confirmed by cryo‐electron microscopy (Cryo‐EM). In addition to verifying the presence of individual nanofibers, Cryo‐EM micrographs showed that the γ‐CD nanotubes tend to align into semi‐crystalline bundles (Figure 3c).

The presence of G inside the nanotube cavity was probed using linear dichroism (LD). Under shear flow, the alignment of the nanotubes and the chromophores within their cavities induced anisotropic absorption (Figure S6). The positive LD spectrum also suggests that the TDM is oriented parallel to the axis of the nanofibers.

Epifluorescence microscopy, enabled by G emission, revealed that concentrated samples form a suspension of uniformly sized rod‐shaped objects (10–20 µm) (Figure 3d). The microscopic rods were further imaged using cross‐polarized optical microscopy (POM), making use of their birefringence (Figure S7). Together, these findings indicate that the secondary assembly of nanotubes, driven by their bundling, leads to a hierarchically structured microcrystalline material, which will hereafter be referred to as rod‐shaped microcrystals.

### 2.4 Stoichiometry of Inclusion Complexes

**FIGURE 4:**
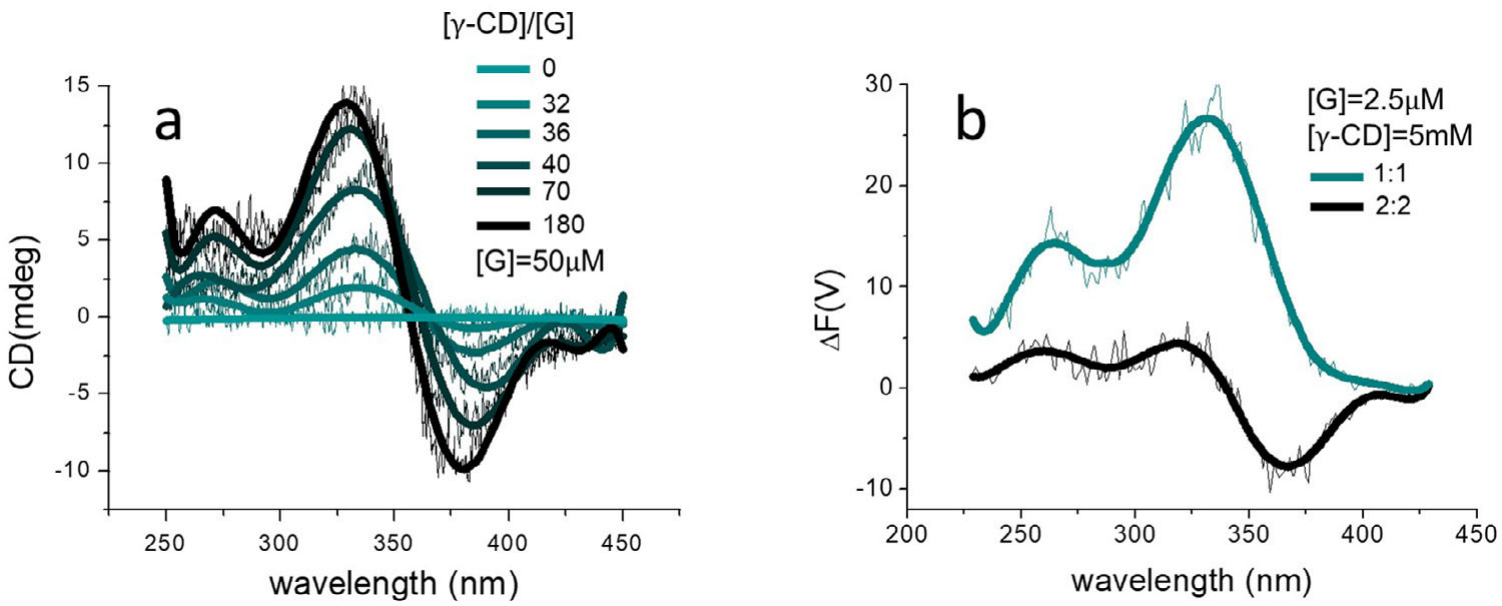
(a) ECD spectra. Induced cotton effect enables selective monitoring of inclusion complexes during binding titrations. (b) FDCD and pathway complexity, sample preparation method influences the stoichiometry of the inclusion complex.

**SCHEME 3:**
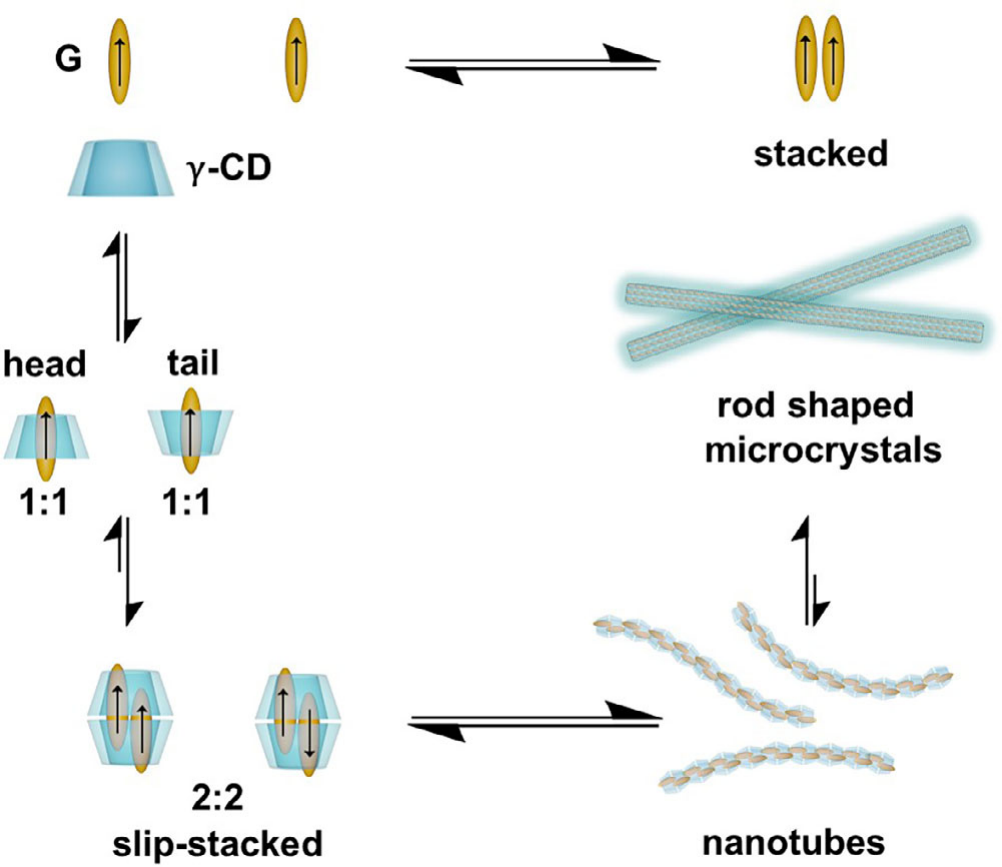
Self‐assembly landscape in the presence of γ‐CD.

Electronic circular dichroism (ECD) was used to investigate the assembly mechanism. When analyzed separately, both G and γ‐CD are spectroscopically silent to ECD; however, increasing the sugar concentration in the presence of G, led to a dichroic signal (Figure 4a). This signal is attributed to the formation of inclusion complexes where the optically inactive chromophore (G), surrounded by the chiral environment of γ‐CD, exhibits an induced Cotton effect. The absence of an isodichroic point during the titration indicates the presence of more than one ECD active species, and following the spectral bands at *λ* = 335 nm and *λ* = 380 nm, it was possible to identify two inter‐dependent but asynchronous binding events (Figures 4a and S8). The ECD spectrum is initially dominated by a positive Cotton band centered at *λ* = 335 nm. As increasing amounts of γ‐CD are titrated, this electronic transition is progressively turned into a negative couplet centered at *λ* = 355 nm. According to Kodaka's rules such transition usually occurs when two chromophores constitute an exciton within the γ‐CD cavity [36], in agreement with the proposed formation of J‐type aggregates during the supramolecular polymerization. The initial positive Cotton signal at *λ* = 335 nm is attributed to the formation of a 1:1 complex, with the guest TDM aligned parallel to the axis of the cyclodextrin host (Scheme 3) [36]. Given the relative sizes of the G (*V*_G_ = 395 Å^3^) and the host's cavity (*V*_γ‐CD_ = 427 Å^3^) [37], the formation of an exciton with a higher stoichiometric ratio in G within the nanotube cavity is unlikely. Consequently, the spectroscopic transition observed in the EDC spectrum is tentatively attributed to the formation of a 2:2 complex, where two chromophores pair in a slipped‐stacked configuration (Scheme 3). This pairing mechanism implies the existence of two distinct species of 1:1 complex, referred to as head and tail (Scheme 3), which are thought to rapidly interconvert and thus remain spectroscopically indistinguishable. As the 2:2 complex accumulates above the CAC, it has been identified as the species responsible for the supramolecular polymerization and is believed to represent the thermodynamic sink of this system (Scheme 3).

This interpretation of the assembly mechanism was verified by fluorescence‐detected circular dichroism (FDCD). The sensitivity of this technique enabled the recording of the ECD spectra of both 1:1 and 2:2 complexes isolated under diluted conditions, confirming the assignment of the Cotton bands in the ECD titration experiment (Figure 4b). The stoichiometry of the complexes varied depending on the method used for sample preparation. For instance, the spectra shown in Figure 4b were recorded at the same concentration of G and γ‐CD, but the 2:2 complex was obtained by diluting samples that had been previously assembled above the CAC. This evidence of pathway complexity supports the greater thermodynamic stability of the 2:2 complex and confirms that this species is responsible for the supramolecular polymerization. It is important to emphasize that the proposed structural assignment is derived from combined absorption, LD, and ECD data, which indicate slipped‐stacked, coaxially aligned chromophores forming an exciton pair. The stoichiometry of the complex is further supported by cavity size considerations, which will be definitively validated by forthcoming x‐ray diffraction (XRD) data.

### 2.5 Geometry of the Inclusion Complex

**FIGURE 5:**
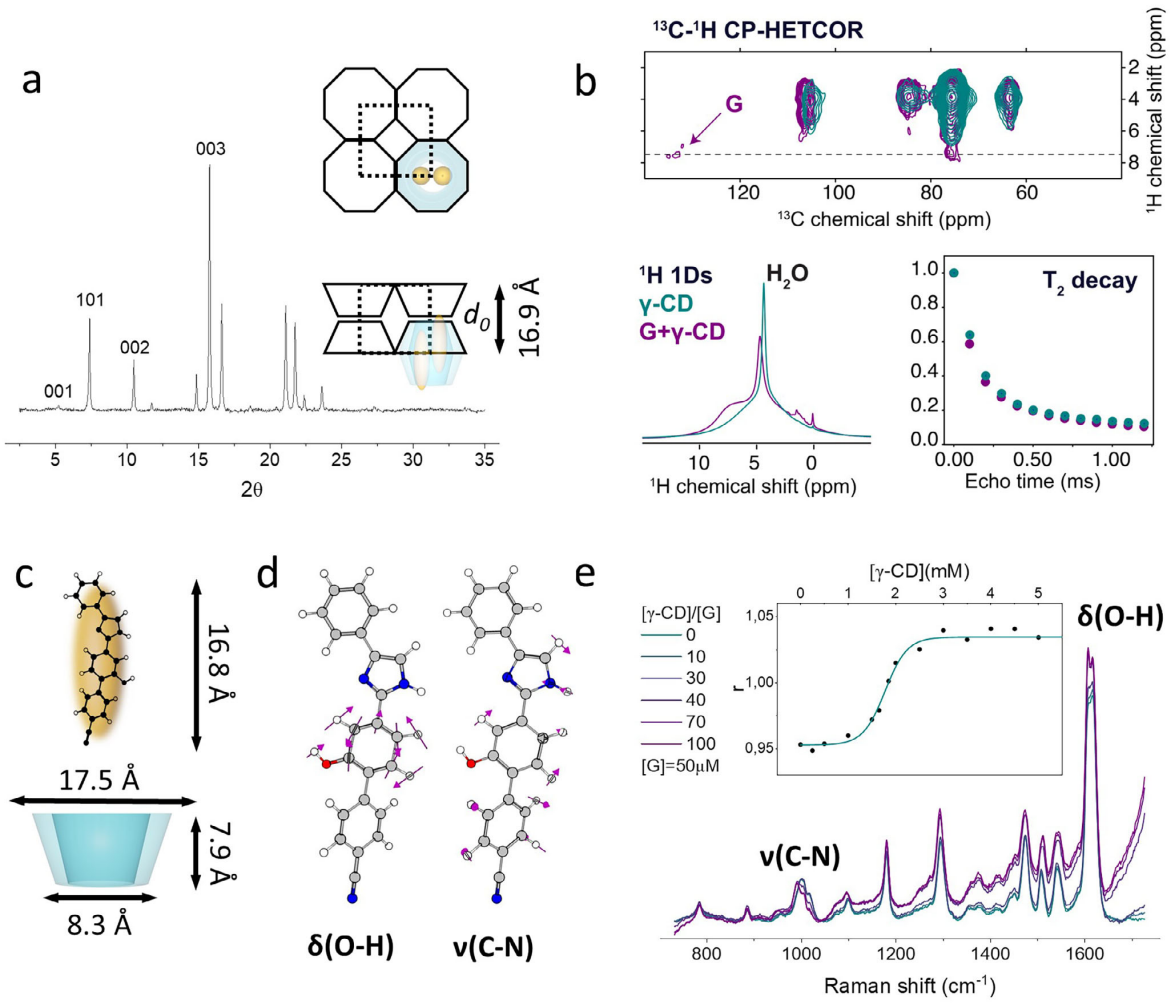
(a) XRD pattern of rod‐shaped microcrystals and extracted unit‐cell parameters. (b) 2D ^13^C‐^1^H and 1D ^1^H ssNMR spectra of γ‐CD (teal) and rod‐shaped microcrystals formed in the presence of G (purple), along with the ^1^H T_2_ relaxation curves. (c) Molecular dimensions of G and γ‐CD. (d) Raman vibrational modes assigned to *δ*(O–H) at 1617 cm^−^^1^ and *ν*(C–N) at 1005 cm^−^^1^. (e) Evolution of the Raman spectrum of G upon inclusion; inset: intensity ratio of the 1607 and 1617 cm^−^^1^ bands as a function of γ‐CD concentration.

The packing mode of the complexes was investigated by XRD. The diffraction from a film prepared by depositing a suspension of rod‐shaped microcrystals onto a silicon wafer shows a well‐defined pattern with a dozen sharp, intense reflections (Figure 5a). Although the organic guest plays a crucial role in determining the packing arrangement, the XRD pattern of the inclusion complexes is typically dominated by cyclodextrin reflections, allowing direct comparison of diagnostic signals with diffraction data reported for other cyclodextrin complexes [38]. Cyclodextrin complexes are generally grouped into three packing modes: cage, layer, and channel. Consistent with the formation of nanotubular fibers, the reflections at 2*θ* = 7.4° and 10.5° are characteristic of a channel‐type packing mode (Figure 5a). Identical reflections have been observed in polymer–cyclodextrin complexes, where linear aliphatic chains such as polypropylene glycol or polyethylene oxide thread through multiple cavities [39, 40], as well as in complexes of fullerene C_60_, which induce a head‐to‐head arrangement of γ‐CDs packed in a tetragonal structure [41]. A second parameter used to interpret the diffraction pattern is the reflection at 2*θ* = 5.2°, typically assigned to the *d*_0_ spacing along the c axis; this signal corresponds to the cavity height of two stacked cyclodextrin units (Figure 5a) [38]. In our case, this 001 peak and its higher‐order 00l reflections at 2*θ* = 10.5° and 15.8° yield a *d*_0_ spacing of 16.9 Å according to Bragg's equation. Strikingly, this value closely matches the molecular length of our guest (*l* = 16.8 Å), confirming that the guest's main axis aligns with the [00l] crystallographic direction, which coincides with the growth axis of the tubular nanofibers (Figure 5a,c). The influence of the guest on the spacing between cyclodextrins indicates that the entire aromatic structure is accommodated in the cavity in a tight‐fitting complex [42], which occupies a tetragonal unit cell with *a* = *b* = 17.5 Å and *c* = 16.8 Å.

Atomic‐level insight into the intermolecular interactions, and evidence for the inclusion of G within the γ‐CD cavities of the crystallites, was obtained by comparing the solid‐state NMR (ssNMR) spectra of lyophilized γ‐CD with those of the rod‐shaped microcrystals formed upon addition of G. The ^1^ ^3^C 1D CP‐MAS spectra of both samples show the major resonances of cyclodextrin (C1, C2/3/5, C4, and C6) (Figure S9). In the presence of G, however, an additional weak and broad signal appears at ∼132 ppm, attributable to aromatic carbons of the guest. However, unresolved signals in the assembled state preclude an unambiguous distinction between parallel and antiparallel chromophore arrangements. Measurable chemical‐shift changes are also detected for the anomeric carbon (C1) and the primary rim (C6) of γ‐CD, indicating altered ring strain and/or specific host–guest interactions (Figure S9). These observations provide direct evidence that the guest is incorporated within the host and remains so after washing and freeze‐drying the colloidal suspensions of crystallites. Further structural insights supporting the inclusion come from the 2D ^1^ ^3^C–^1^H CP‐based HETCOR spectrum (Figure 5b). In addition to the γ‐CD peaks, a correlation marked with an arrow is observed at ∼132 ppm (^1^ ^3^C) and 7.3 ppm (^1^H), which arises from the guest. The contact time (1 ms) is sufficiently long to probe medium‐range intermolecular proximities; the appearance of a weak cross‐peak, highlighted by a dashed line in the spectrum connecting 7.3 ppm (^1^H) with the C2/3/5 carbon frequency (∼76 ppm), supports spatial interactions between the guest and the γ‐CD's secondary rim and interior cavity. Regarding the presence of solvent molecules in the inclusion complexes, the ^1^H 1D MAS spectra of both samples—despite lyophilization—are dominated by a relatively broad resonance at 4.5–5 ppm, assigned to residual bound water (Figure 5b). Upon guest inclusion, this signal undergoes a small but measurable downfield shift (4.4 → 4.7 ppm), typically interpreted as evidence of stronger hydrogen bonding. The ^1^H *T*_2_ spin–spin relaxation times were determined by Hahn‐echo experiments to probe the mobility of this water (Figure 5b). These experiments showed very short decay times (< 0.5 ms), fully consistent with bound water. By contrast, highly mobile bulk water would be expected to exhibit much longer *T*_2_ values [43].

These results, together with the evidence for J‐type guest pairs, suggest that interfacial water may bridge proton‐donor and acceptor sites brought into proximity by the slip‐stacked configuration induced by the cavities. Raman spectroscopy was used to study the effects of intercalation on the vibrational spectra of G with the aim of identifying interactions between specific functional‐groups and possible signatures of an intermolecular hydrogen bond within the guest pair. Using excitation at a wavelength resonant with the electronic absorption of the guest provides for resonance enhancement of the Raman scattering. The enhancement is sufficient to enable recording of the vibrational spectra of G at concentrations used in the titration experiments monitored by ECD. The Raman scattering of the cyclodextrin is not enhanced and is too weak to be observed significantly in the spectra (Figure 5e).

The Raman spectra of G show several changes in intensity and Raman shift upon titration with γ‐CD, with the most pronounced features observed at 1611 cm^−^^1^ and in the 990–1100 cm^−^^1^ region (Figure 5e). According to the simulated Raman spectrum (Figure S10a–c), the intense band centered at 1611 cm^−^^1^ arises from two overlapping aromatic stretching modes. Although both are skeletal vibrations, one is primarily localized on the nitrile‐bearing ring, while the other is dominated by the C = C stretching and O–H in‐plane bending vibrations localized on the central phenolic ring (Figures 5d and S10d). Experimentally, this band progressively splits into two features at 1607 and 1617 cm^−^^1^, that become increasingly well resolved during the titration, consistent with a change in conformation and reduced degrees of freedom of the aforementioned functional groups induced by the cavities (Figure 5e). Furthermore, the ratio of intensities at 1607 and 1617 cm^−^^1^ (*r* = *I*_1607_/*I*_1617_) follows a sigmoidal dependence on [γ‐CD], with an inflection at 1.8 mM—in excellent agreement with the ECD data—confirming that the spectral changes are caused by the intercalation of the central ring bearing the photoacidic group during the formation of the complex (Figure 5e).

Additional spectral changes are observed for two broad bands at 1005 and 1100 cm^−^^1^ (Figure 5e). According to DFT calculations and previous reports, these correspond to C–N stretching coupled with C–H in‐plane bending and deformation of the imidazole ring (Figures 5d and S10a–c,e) [44]. The broad line shapes suggest hydrogen‐bond involvement. Upon formation of the complex, the 1005 cm^−^^1^ band is replaced by a new feature at 992 cm^−^^1^, while the 1100 cm^−^^1^ band shifts to 1096 cm^−^^1^. Both modes are recognized tautomeric markers for histidine in aqueous solution, being sensitive to imidazole protonation [44]. Finally, the shoulder at 1018 cm^−^^1^, assigned to out‐of‐plane C–H twisting of the peripheral aromatic ring adjacent to the imidazole, disappears upon titration, suggesting that out‐of‐plane motions are restricted by stacking of the guest within the confined γ‐CD cavity (Figure S10f).

This additional characterization reinforces the structural model: XRD and ssNMR confirm a tight inclusion complex while excluding partial intercalation. The evidence of the bound nature of water in the complexes, together with the changes in the vibrational signatures of the phenolic and imidazole groups, reflect the altered hydrogen bonding and conformational restriction induced by the assembly.

### 2.6 ESPT Mechanism in Nanotube Confinement

**FIGURE 6:**
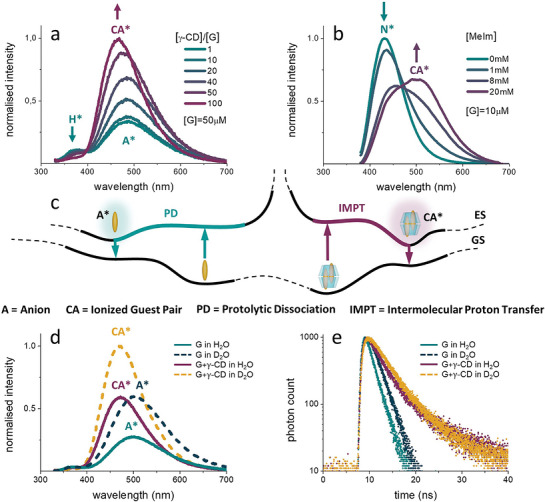
(a) Changes in emission upon supramolecular polymerization in the presence of γ‐CD. (b) Changes in emission upon titration with methyl imidazole in dichloromethane. (c) Mechanistic transition from ESPD to intermolecular ESPT (ESIMPT) induced by pre‐organization of the guest pair in the nanotube cavities. (d) Fluorescence emission, isotope effect. (e) Emission lifetimes determined by TCSPC, isotope effect.

The effect of supramolecular polymerization on the photophysical behavior of G was studied by analyzing its fluorescence emission in the presence of γ‐CD. Increasing the concentration of γ‐CD across the CAC, the main ESPT band experienced up to a five‐fold increase in intensity and a slight hypsochromic shift of its emission peak (Figure 6a). The increase in the fluorescence anisotropy (rA* = 0.1, rCA* = 0.23) observed during the cyclodextrin titration is compatible with the confinement of G within the nanotubes cavities, which is expected to restrict the tumbling of the emitter. As the equilibrium shifts towards the formation of the nanotubes the shoulder band assigned to H* emission was suppressed, confirming that supramolecular polymerization drains the free G emitter from the assembly equilibrium (Figure 6a, Scheme 3).

The transition to a hydrophobic micro‐environment accounts for the subtle shift in the ESPT band [45]. However, the observed increase in the quantum yield of emission (ϕA* = 0.05, ϕCA* = 0.11) is unusual for photoacids in confinement (Figure S11a,c) [9]. Typically, water nanopools in cyclodextrin cavities rarely support PT and tend to suppress ESPD emission by promoting the geminal recombination and emission from protonated state [10]. The effects of cyclodextrin compartmentalization were assessed by comparing the spectral changes in our emitter with those of five known Brønsted photoacids, revealing a markedly different behavior (Figure S12a–f). Unlike our system, confinement in all other cases enhances the characteristic blue‐shifted band of the protonated form. None of the reported examples shows our robust enhancement of the ESPT band. Instead, ESPT emission is either unaffected or, more commonly, decreases in intensity due to equilibrium partitioning between free and confined states. This comparison indicates that a generic cavity effect is insufficient to explain the observed emission changes.

This contrasting behavior suggests a potential shift in the ESPT mechanism of G during supramolecular polymerization in the presence of γ‐CD, distinct from both its monomeric G(A*) and self‐aggregated (H*) emission pathways in water. Our hypothesis is that the formation of the J‐type aggregates within the nanofibers involves the pre‐organization of a hydrogen bond between the phenol and imidazole sites of neighboring emitters, possibly mediated by interfacial water, enabling ESPT in confinement to occur intermolecularly within the guest pairs 2G(CA*) (Figure 6c, Scheme 1). The role of the imidazole site was investigated by titrating G with an external base in dichloromethane. As noted, in the absence of an external acceptor, G exhibits only a blue‐shifted band from its protonated form (Figure S2). Addition of methyl imidazole causes this band to decrease as a red‐shifted ESPT band emerges (Figure 6b). This confirms that an accessible imidazole acceptor can enable ESPT in hydrophobic conditions, supporting the assignment of the confinement‐induced red‐shifted band to intermolecular ESPT involving a co‐confined acceptor.

The solvent isotope effect on quantum yield and fluorescence lifetime was investigated to provide deeper insight into the relaxation mechanism within the nanotube confinement. Following hydrogen/deuterium exchange between G and the solvent, the kinetic isotope effect is expected to quench the emission of the photoacid—provided that the relaxation pathway involves an ESPT mechanism [46]. On the other hand, the slower vibrational relaxation of G in deuterated water should increase its photoluminescence [47, 48, 49]. These two effects counteract each other, affecting both quantum yield and lifetime of emission, with the microenvironment potentially determining which contribution predominates.

Exponential decay fitting of the emissions from monomers G(A*) and H‐aggregates (H*) in H_2_O revealed two distinct lifetimes (*τ*_A*_ = 0.3 ns, *τ*_H*_ = 1.2 ns), which is consistent with the H* band originating from the formation of an H‐type exciton (Figures 6d,e and S12g, Table 1). In D_2_O, the quantum yield of the ESPT band G(A*) more than doubled compared to H_2_O (ϕ_D_(A*)/ϕ_H_(A*) = 2.5). The fluorescence lifetime also increased, as evidenced by a change in the slope of the exponential decay, although it remained in the sub‐nanosecond range (*τ*_A*_ = 0.9 ns) (Figure 6d,e).

**TABLE 1:** Emission lifetimes from TCSPC data fitting.

Emitter^a^	*τ*_1_ (ns)	%^b^	*τ*_2_ (ns)	%	*χ* ^2^
H* (H_2_O)	1.2				0.97
H* (D_2_O)	1.5				0.96
A*(H_2_O)	< 1				2.00
A*(D_2_O)	< 1				1.12
CA*(H_2_O)	< 1	38	7.2	62	1.19
CA*(D_2_O)	1.0	47	4.5	53	1.04

^a^ H* emission was collected using a 390 ± 10 nm band pass filter, A* and CA* emission, using a 500 nm long pass filter.

^b^ The % of the biexponential decays are based on the fractional intensities of the positive decay components.

Upon γ‐CD addition and supramolecular polymerization, the decay of the main emission band becomes biexponential, presenting a sub‐nanosecond component associated with a relaxation of G(A*) from the free monomers in solution, and a long‐lived component (*τ*_2_ = 7.2 ns), assigned to the relaxation of a ionized guest pair 2G(CA*) confined in the nanotubes (Figure 6d,e, Table 1).

Opposite isotope effects were observed on the two components of the decay. While the emission lifetime from G(A*) increased in deuterated water, as observed in the previous experiment, the lifetime of confined emitters nearly halved (*τ*_2_ = 4.5 ns) (Table 1). This reversed trend is reflected in the quantum yield of emission, which overall increases (ϕ_D_(CA*)/ϕ_H_(CA*) = 1.6) because of the contribution of G(A*) to the biexponential decay, though to a lesser extent (−36%) with respect to that observed in the absence of γ‐CD (Figure 6d).

These experimental results indicate that in hydrophobic nanocavities, where G is poorly hydrated, solvent‐assisted vibrational relaxation is less significant than the kinetic isotope effect. The distinct microenvironments of free versus confined G emitters explain the opposite isotope effects observed for the two components of the biexponential decay in assembled samples. Most importantly, the reduction of the longer lifetime component, *τ*_2_—attributed to the emission from the confined guest pair 2G(CA*)—indicates a pronounced primary kinetic isotope effect, confirming that radiative relaxation in the nanocavities occurs via intermolecular ESPT mechanism.

### 2.7 Photostability and Anisotropic Emission

**FIGURE 7:**
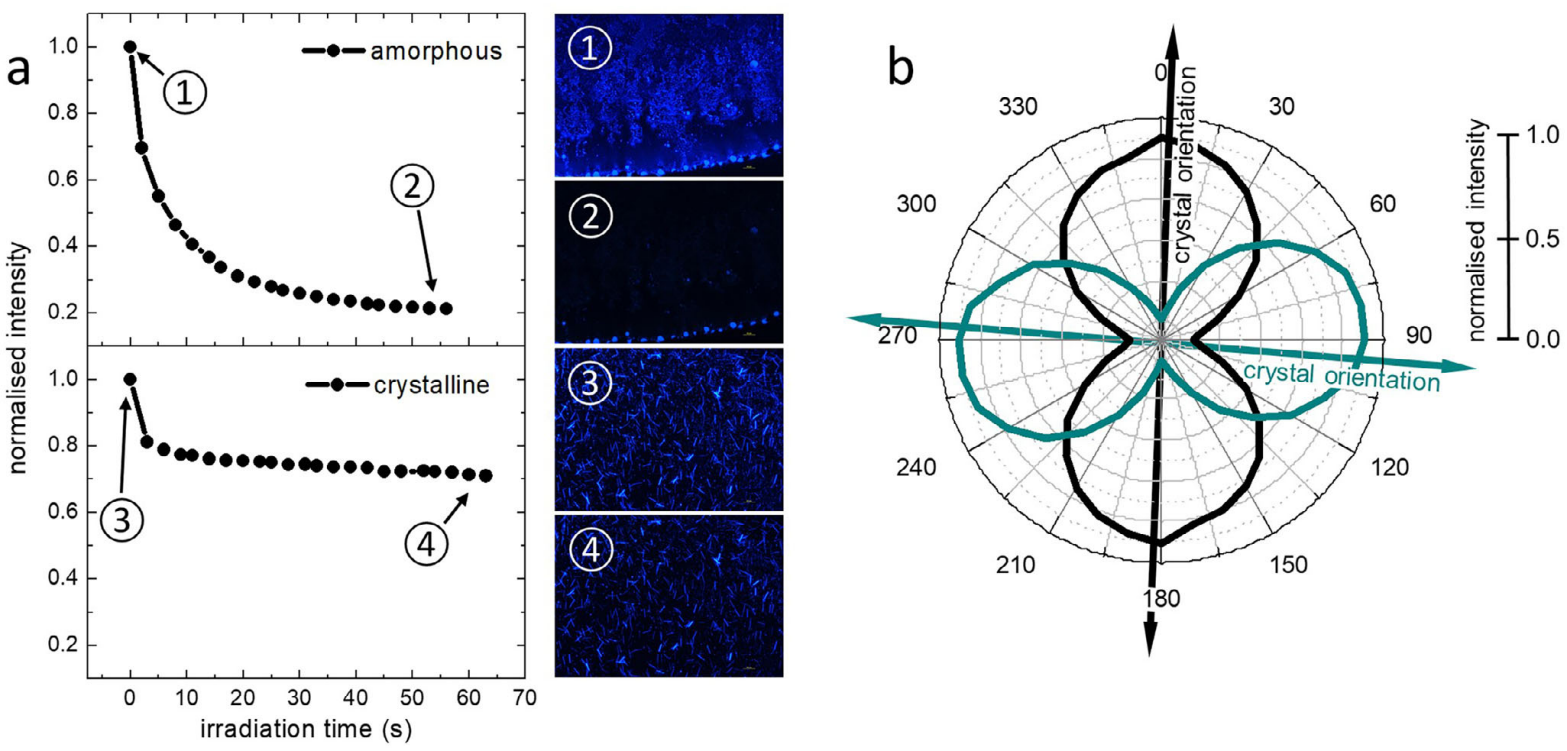
(a) Analysis of epifluorescence micrographs comparing the photobleaching in amorphous samples and rod shaped microcrystals. (b) Polarized emission intensity from a pair of orthogonally oriented rod shaped microcrystals.

The mechanism of photo‐relaxation of the emitter often influences the optical properties of the material resulting from its self‐assembly. Epifluorescence microscopy was used to investigate the photoluminescence of the rod shaped microcrystals formed upon co‐assembling of G with γ‐CD. Analysis of emission intensity during prolonged photoexcitation revealed that, compared to amorphous deposits of G, rod‐shaped microcrystals exhibited significantly greater resistance to photobleaching (Figure 7a). The enhanced photostability induced by the supramolecular host indicates that the template effect effectively suppresses alternative relaxation pathways, other than ESPT, that could lead to photodegradation [5].

Under isotropic illumination, the emission intensity of the crystallites varied with the analyzer orientation, following a dipolar pattern and reaching a maximum when the analyzer was aligned with the main axis of the rod‐shaped microcrystals (Figure 7b). The photoluminescence anisotropy is consistent with inclusion complexes in which the molecular alignment of guest pairs is preserved with nanotube bundling showing long‐range crystalline order.

## 3 Conclusion

Confinement of an ESPT emitter in a supramolecular polymer induces a mechanistic switch in PT. Despite being amphoteric, the photoacid undergoes ESPD in water, with H‐type self‐aggregation quenching its emission. In contrast, co‐assembly with γ‐CD to form inclusion complexes promotes ESPT emission from nanotubes formed by supramolecular polymerization. The mechanism for self‐assembly reveals that excited state intermolecular PT is enabled by an optimal pre‐organization of the proton donor and acceptor sites within the hydrophobic micro‐environment provided by the nanotubes.

Spectroscopic and diffraction data show that these nanocavities template the formation of a tight‐fitting complex in which fully intercalated emitters pair in a slipped‐stacked configuration, bringing neighboring proton‐donor and proton‐acceptor sites into close proximity. Changes in the vibrational frequencies of the photoacidic phenol and the imidazole ring further support the formation of an intermolecular hydrogen bond connecting these two sites.

The primary kinetic isotope effect, observed in the emission lifetime after supramolecular polymerization indicates that the photochemical pathway of confined emitters involves ESPT. However, the enhanced ESPT band and the absence of protonated‐form emission upon confinement—contrary to the typical trend observed for Brønsted photoacids—is incompatible with the ESPD mechanism observed in the absence of γ‐CD.

Together, these findings indicate a mechanistic switch from ESPD to intermolecular ESPT induced by a supramolecular templating effect. Integrating structural characterization with the photophysical effects of self‐assembly, the proposed mechanism represents the most parsimonious interpretation consistent with the collective data.

Despite the low level of hydration within the supramolecular polymer, we do not exclude the possibility that interfacial water molecules can mediate PT. However, the effects observed in the photoluminescence of the emitter require coordination with a basic site that stabilizes the transferred proton in the excited state. The conformation of the nanocavities and their microenvironment provide this specific balance of interactions, pre‐organizing the proton donor and acceptor sites and overcoming the hydrogen‐bonding competition from bulk water.

The switch in the excited state pathway is manifested in the optical properties of the crystallites formed upon assembly, highlighting the potential of this approach for integrating photoacid emitters within supramolecular polymer‐based materials. The ability to direct PT by confinement in supramolecular assemblies represents a promising strategy for developing general acid‐catalyzed reactions in nanoreactors, thus rivaling the domain of enzymatic catalysis.

## Author Contributions

**Luis C. Pantaleone**: conceptualization, methodology, investigation, writing – original draft, formal analysis, writing – review and editing. **Robert Hutchings**: investigation, writing – review and editing. **Bente Reus**: investigation, writing – review and editing. **Jacopo Martinelli**: investigation, formal analysis. **Alessia Lasorsa**: investigation, writing – review and editing, formal analysis. **Patrick C.A. van der Wel**: writing – review and editing, formal analysis. **Marc C. A. Stuart**: writing – review and editing, investigation, methodology, formal analysis. **Wesley R. Browne**: writing – review and editing, formal analysis, investigation. **Tibor Kudernac**: conceptualization, formal analysis, supervision, funding acquisition, project administration, writing – review and editing.

## Conflicts of Interest

The authors declare no conflicts of interest.

## Supporting information



The authors have cited additional references within the Supporting Information [50, 51, 52, 53, 54, 55, 56, 57, 58, 59, 60, 61, 62, 63, 64, 65, 66, 67, 68, 69, 70, 71]. **Supporting File**: anie72931‐sup‐0001‐SuppMat.pdf.

## Data Availability

The data that support the findings of this study are available from the corresponding author upon reasonable request.
