# Supplementary material for: Redirecting Excited‐State Proton Transfer Through Supramolecular Polymerization in Nanoconfinement
Source: Angew Chem Int Ed Engl. 2026 Jun 8;65(32):e7594145. doi: 10.1002/anie.7594145 (PMC13427185; doi:10.1002/anie.7594145)
Supplement: Supplementary file 1 — The authors have cited additional references within the Supporting Information [50, 51, 52, 53, 54, 55, 56, 57, 58, 59, 60, 61, 62, 63, 64, 65, 66, 67, 68, 69, 70, 71]. Supporting File: anie72931‐sup‐0001‐SuppMat.pdf. [file ANIE-65-e7594145-s001.pdf]

Supplementary information for: “Redirecting Excited State Proton Transfer through Supramolecular Polymerization in Nanoconfinement”

Luis C. Pantaleone,* Robert Hutchings, Bente Reus, Jacopo Martinelli, Alessia Lasorsa, Patrick C.A. van der Wel, Marc C. A. Stuart, Wesley R. Browne, Tibor Kudernac*

Table of contents

1. Materials and Methods……S2

1.1 Materials and Methods……S 2

2. Supplementary Figures……S4

2.1 Supplementary Figure 1: Spectroscopic titrations……S 4

2.2 Supplementary Figure 2: Solvatochromism ……S 5

2.3 Supplementary Figure 3: Effect of Chaotropic salt ……S 5

2.4 Supplementary Figure 4: Excitation spectra ……S 6

2.5 Supplementary Figure 5: Supramolecular polymerization……S 6

2.6 Supplementary Figure 6: Linear dichroism……S 7

2.7 Supplementary Figure 7: Cross polarized optical microscopy……S 7

2.8 Supplementary Figure 8: Characterization of inclusion complexes…… S 8

2.9 Supplementary Figure 9: Solid state NMR……S 8

2.10 Supplementary Figure 10: Raman spectroscopy……S 9

2.11 Supplementary Figure 11: Quantum yield ……S 10

2.12 Supplementary Figure 12: Effects of compartmentalization and lifetime of emission……S 11

3. Synthesis and Characterization……S 12

3.1 Synthesis of 4'-formyl-2'-hydroxy-[1,1'-biphenyl]-4-carbonitrile (1)……S 12

3.2 Synthesis of G……S 14

4. Computational Methods……S 16

4.1 Computational Methods……S 16

5. References……S 17

5.1 References……S 18

1. Materials and Methods

Chemicals and solvents were obtained from commercial sources and used without further purification unless stated otherwise.

Photoacid G was prepared in a two-step linear synthesis consisting of a Suzuki-Miyaura cross-coupling reaction between 3-hydroxy-4-iodobenaldehyde and 4-cyanophenyl boronic acid, followed by a Debus-Radziszewski imidazole synthesis. The final product was characterized by ^{1}H NMR, ^{13}C NMR, HRMS, UV/vis absorption, FTIR and Raman spectroscopy. Details of synthesis and characterization are provided in section 3 of this document.

Procedure for the preparation of nanotube fibres was based on the solvent processing method.[1] A stock solution of guest in methanol ([G]=5 mM) was dispersed in an aqueous solution of γ-cyclodextrin ([γ-CD]=5 mM) adjusting the final concentration of G to 50 µM (water/methanol 99:1). After the addition, samples were stirred using a vortex mixer for a few seconds and stored at room temperature for 30 minutes while supramolecular polymerization proceeded. Stock solutions were freshly prepared for each experiment to avoid pre-aggregation of G.

Procedure for the preparation of rod shaped microcrystals was based on the same processing method used for preparing the suspensions of nanotube fibres. A stock solution of G ([G]=5 mM) in methanol was dispersed an in aqueous solution of γ-cyclodextrin ([γ-CD]=10 mM), adjusting the final concentration of G to 0.5 mM (water/methanol 9:1). After the addition, samples were stirred using a vortex mixer for a few seconds and stored at room temperature for 30 minutes while supramolecular polymerization proceeded obtaining a colloidal suspension of rod shaped microcrystals. Stock solutions were freshly prepared for each experiment to avoid pre-aggregation of G.

Procedure for the preparation of cast films of microcrystals. Colloidal suspensions of rod-shaped microcrystals were cast onto glass slides (for microscopy) or onto silicon wafers (for XRD). After 30 minutes of deposition, the dispersant was blotted to remove the excess of γ-CD in solution, and the films were dried under reduced pressure.

Procedure for the preparation of lyophilized powders of microcrystals. Colloidal suspensions of rod-shaped microcrystals were concentrated by centrifugation (20 °C, 10^4 rcf, 15 min), and the resulting pellets were washed three times with Milli-Q water. The microcrystals were then resuspended and freeze-dried to obtain lyophilized powders.

Spectroscopic titrations. pH titrations were carried out by addition of HCl or NaOH solutions to adjust pH, determined using a Mettler Toledo FiveEasy F-20 benchtop pH/mV meter equipped with a pH electrode inLab microprobe. For pH titrations outside the calibration range of the instrument (pH < 2) the pH was calculated from the [HCl]. The absorption data were processed using the optical spectroscopy software Spectragryph. pKa values were determined from the inflections points obtained following sigmodal fitting of the change in absorption with pH. Except for the spectroscopic data from the pH-titration experiments—where the pH is indicated on each spectrum—all other spectroscopic measurements presented in the manuscript were recorded at neutral pH.

UV/vis absorption spectra were recorded on an (Analytik Jena) Specord 210 Plus spectrophotometer spectropolarimeter at 20 °C in a 1 cm path length quartz cuvette.

Electronic Circular Dichroism (ECD) spectra were recorded with a (JASCO) J-815 spectropolarimeter at 20 °C in a 1 cm path length quartz cuvette.

Linear Dichroism (LD) spectra were recorded using a (JASCO) J-815 spectropolarimeter with a micro-volume Couette flow cell (Dioptica Scientific Ltd.), consisting of coaxially aligned stationary quartz rod (D = 2.5 mm) and rotating quartz cylinder (ID = 3 mm), rotating at 3000 rpm at 20 °C. Deionised water was used as reference.

Emission and excitation spectra were recorded with an FS5 Spectrofluorometer (Edinburgh Instruments). For fluorescence anisotropy, excitation and emission arms were equipped with calcite polarizing prisms. In a typical fluorescence titration experiment the absorbance of the sample was maintained between 0.2-0.3 at the excitation wavelength used (λ_{ex} 320 nm). The quantum yield of emission of G(N) monomers was determined by Williams' method using $[Ru(bpy)_3](PF_6)_2$ as a comparative standard,[2] and an excitation wavelength of λ_{ex} 360 nm to avoid co-excitation of H aggregates. Quantum yield ratios were determined by comparing the integrated emission intensities, corrected for the optical densities at the excitation wavelength.[3]

Fluorescence-detected Circular Dichroism (FDCD) measurements were performed on a (JASCO) J-815 spectropolarimeter equipped with accessory (JASCO) FDCD-405. The PMT detector was attached orthogonal to the excitation path with a long-pass filter (λ ≤ 380 nm) placed before the PMT detector unit. The signal was collected at fixed HT (400 V) and 50 nm/min scan speed, in 1 cm path length quartz cuvettes at room temperature.

Fluorescence decay lifetimes were measured using a Picoquant 300 TCSPC: a PicoQuant PDL 800-B diode laser driver was connected to a PicoQuant PLS 255 nm led-head equipped with 250-350 nm bandpass filter. A PicoQuant Tau-SPAD-100 single photon counting module equipped with a 390±10 nm band pass or 500 nm long pass filter was connected with a PicoQuant PicoHarp 300 TC-SPC module. The Tau SPAD was powered by a DSN 102 dual SPAD power supply. The internal trigger from the PDL was used as input for time stamping on the PicoHarp. Samples were held in a Thorlabs cuvette holder CVH100 with CVH100-CV lid. Data were fit using a FluoFit, the IRF used was generated by scattering from water, and Rhodamine 6G was used as a standard for validation of the method (Figure S-11). For the experiments on kinetic isotope effect the deuteration of the G acidic protons was obtained by $^{1}H/^{2}H$ exchange with deuterated water (isotropic purity ≥99.9%) used in the preparation of the sample.

Epifluorescence microscopy was performed in a Nikon Eclipse LV100N POL microscope equipped with a 12V-50W halogen lamp for diascopic illumination as well as a 120V-130W C-LHGFI HG lamp mounted on intenselight C-HGFI fiber illumination system for episcopic illumination. Sample preparation followed the procedure described above for cast films. For the epifluorescence measurements the microscope was equipped with a UV-2A filter (bandpass excitation λ_{ex} 330-380 nm, long pass > 420 nm). An analyzer was mounted between the epi illuminator module (LV-UEPI) and the camera for measurement of the anisotropy of photoluminescence.

XRD: Sample preparation followed the procedure described above for cast films. The X-ray diffraction measurements were carried out using a Bruker D8 Advance diffractometer equipped with a Cu-Kα source. The cell parameters were extracted from the diffraction pattern using N-TREOR09 from the software EXPO2014.[4]

Solid-state NMR experiments were performed on a Bruker AVANCE NEO 600 MHz (14.1 T) spectrometer equipped with a 3.2 mm EFree HCN MAS probe. Samples were packed into 3.2 mm thin-wall zirconia rotors using bottom- and top-insert spacers. Approximately 8 mg of γ-CD and 3 mg of rod-shaped microcrystals (γ-CD/guest) were used for the analysis of the solid samples. Sample preparation followed the procedure described above for lyophilized powders. Experiments were carried out at a MAS spinning rate of 20 kHz and a temperature setpoint of 288 K. ^{1}H MAS NMR spectra were acquired using a ^{1}H 90° pulse of 2.5 µs (rf field strength: 100 kHz), a recycle delay of 4 s, and 16 scans. Echo-detected experiments with rotor synchronization were performed with a recycle delay of 10 s and 8 scans, with the total echo time incremented from 0 to 1.2 ms. ^{13}C cross-polarization (CP) spectra were recorded using a ^{1}H 90° pulse of 2.5 µs (rf field strength: 100 kHz), a ^{13}C nutation frequency of 50 kHz (5 µs 90° pulse), 0.018 s acquisition time, 4 s recycle delay, and 2k scans. A 70–100% ramped $^{1}H \rightarrow ^{13}C$ CP pulse with a contact time of 1 ms was used. During acquisition, TPPM (Two-pulse Phase-modulated) ^{1}H decoupling at 100 kHz was applied.[5] Two-dimensional ^{13}C–^{1}H HETCOR spectra were acquired using frequency-switched ^{1}H homonuclear Lee–Goldburg (FSLG) decoupling during the indirect dimension (t_1). CP with 1 ms contact time was employed for $^{1}H \rightarrow ^{13}C$ magnetization transfer, using a ^{13}C rf amplitude of ~50 kHz, 3.5 s recycle delay, and a total of 78 increments with an increment time of 75.5 µs. 256 scans were collected for each increment for the γ-CD sample and 512 for the rod shaped microcrystals (γ-CD/guest) complex. A ^{1}H rf field strength of 100 kHz was used for FSLG decoupling. TPPM ^{1}H decoupling during acquisition was ~100 kHz. Spectra were acquired with Bruker TopSpin, processed using NMRPipe, and visualized with CcpNmr Analysis.[6-7] ^{1}H and ^{13}C chemical shifts were indirectly referenced to aqueous DSS using external calibration to the ^{13}C resonances of adamantane (left-most peak set to 40.49 ppm).[8]

Raman spectroscopy in solution: Raman spectra at 355 nm (Cobolt Zouk, 10 mW) were recorded in 180° backscattering arrangement in 1 cm pathlength quartz cuvettes. The laser was brought into the optical axis of the spectrometer with a dichroic mirror (Semrock) and focused on the sample with a 7.5 cm planoconvex lens which also collected and collimated the backscattered Raman scattering. The Raman scattering passed through the dichroic and a Rayleigh line rejection filter (Semrock) before being focused into a Shamrock500i spectrometer equipped with a 1800 l/mm grating blazed at 300 nm and a idus-420-BU CCD camera by a 10 cm planoconvex lens.

Solid state Raman and FTIR spectra: Raman spectroscopy at 785 nm was performed with an Olympus BX51M microscope equipped with a fibre-coupled laser (BT785, ONDAX, 500 mW) and a fibre coupled Andor Shamrock SR-163 spectrograph and Andor iVac 316 DR-316B-LDC-DD CCD camera, Spectra were acquired with Andor Solis and calibrated with polystyrene (SI-14). The FTIR spectra were recorded on an Agilent Cary 630 FTIR spectrometer (SI-14).

Cryo-Electron microscopy images were recorded with a Tecnai T20 TEM microscope (FEI) operating at 200 keV using a Gatan model 626 cryo-stage sample holder.

Dynamic light scattering (DLS) measurements were carried out with a Zetasizer Ultra Red (ZSU3305) from Malvern Panalytical.

2. Supplementary Figures

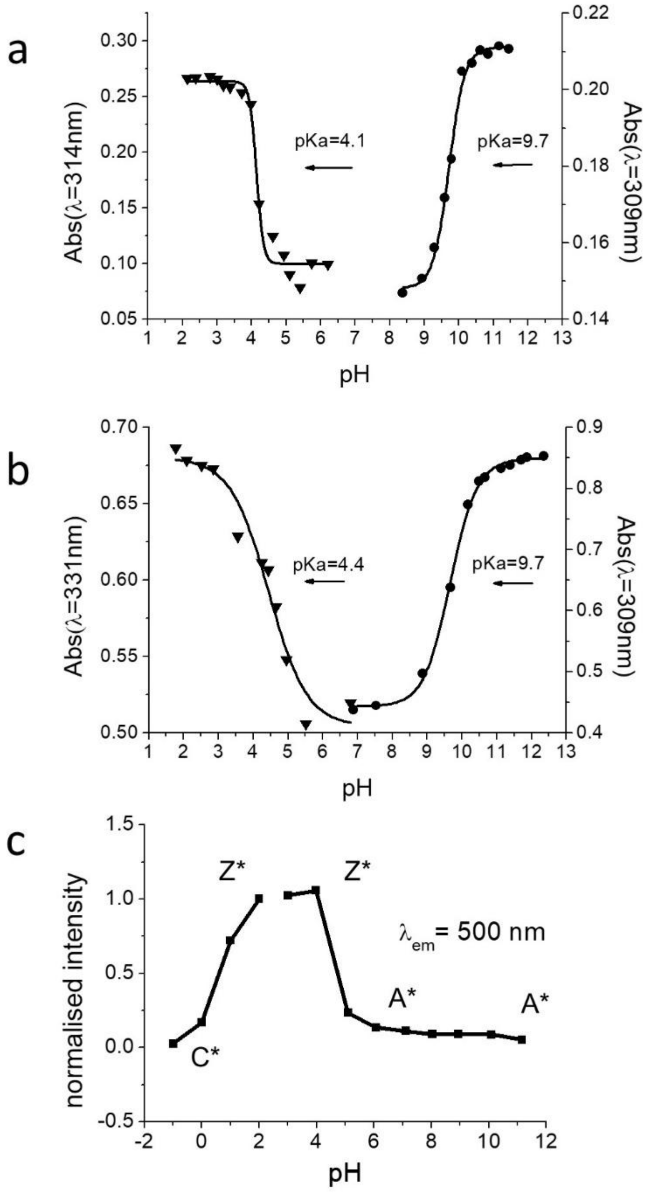

Figure S1: Spectroscopic titrations. (**a**) Absorption of G vs pH. (**b**) Absorption of G vs pH in the presence of γ-CD. (**c**) Emission intensity of G vs pH.

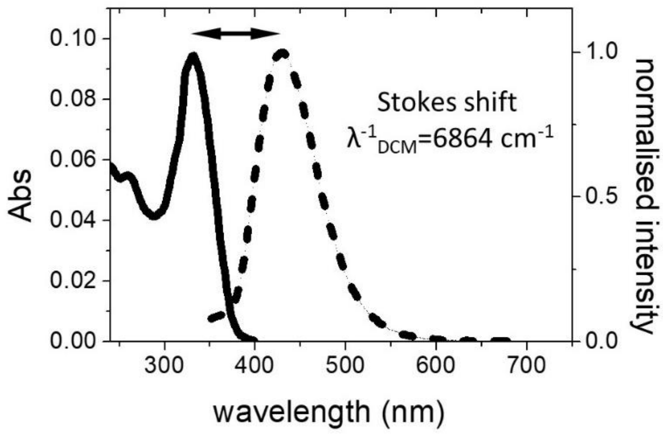

Figure S2: Solvatochromism. Absorption and Emission spectra of G in dichloromethane.

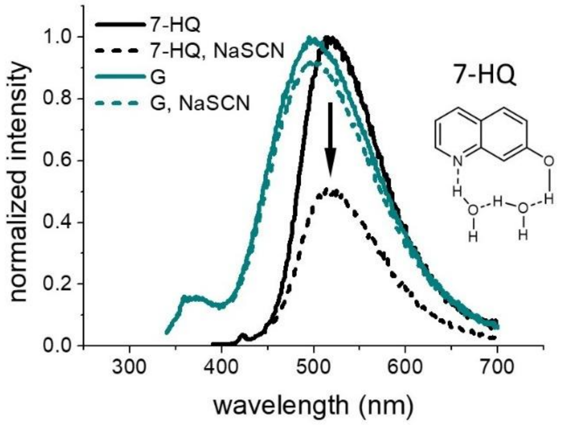

Figure S3: Effect of chaotropic salt. Comparing the effect of sodium thiocyanate ([NaSCN]=100mM), on the intensity of emission of (7-HQ) and G in water (pH=7±0.5).

Effect of chaotropic salt: It has been reported that 7-hydroxyquinoline (7-HQ) and related photoacids can transfer protons to distant sites via the formation of extended proton wires, networks of hydrogen bonded water molecules that bridge the proton donor and acceptor.[9-10] While all ESPT processes require pre-organised hydrogen bonds, proton wires specifically rely on a well-structured and continuous hydrogen-bonded network. To test for the presence of such structures, we added sodium thiocyanate (NaSCN), a chaotropic salt known to disrupt water's hydrogen-bonding network.[11] As expected, the emission of 7-HQ was significantly quenched, consistent with the disruption of its proton wire. In contrast, the emission of **G** remained largely unaffected under the same conditions (Figure S3), suggesting that proton transfer in **G** does not depend on an extended water wire between phenol and imidazole groups.

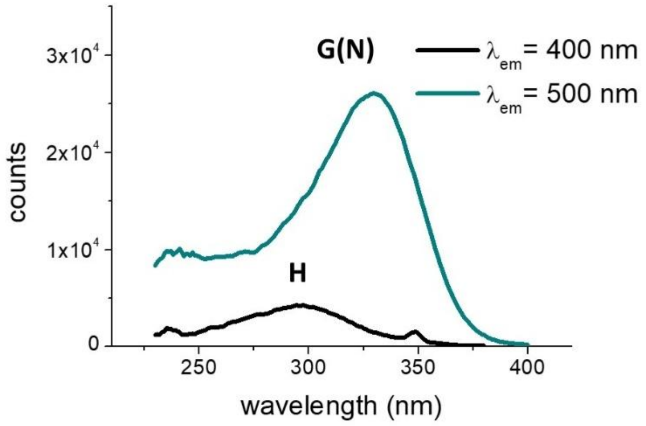

Figure S4: Excitation spectra. Excitation spectra of G(N) and G(H) emission bands in water (pH=7±0.5).

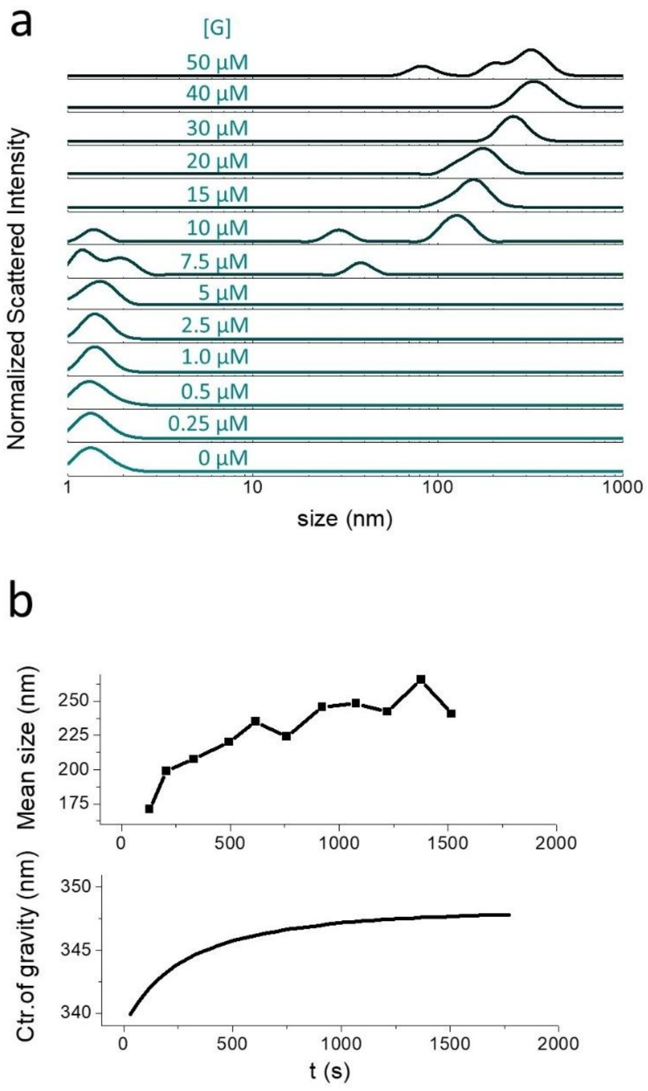

Figure S5: Supramolecular polymerization. (**a**) Distributions of normalized scattered intensities from DLS measurements. For CAC determination, mode values were taken from the population with the largest aggregate size when multiple populations present. (**b**) Polymerization of nanotubes monitored by DLS (top) and by bathochromic shift in the absorption spectra (bottom).

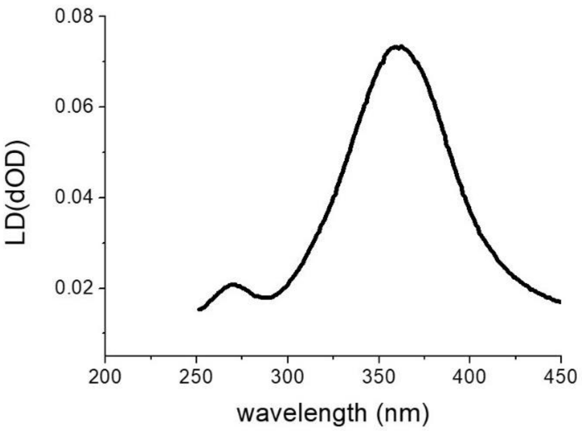

Figure S6: Linear dichroism. Linear dichroic absorption spectra of G recorded upon alignment of nanofibers in the flow generated by spinning the LD cell probe ([G]=50µM; [γ-CD]=5mM).

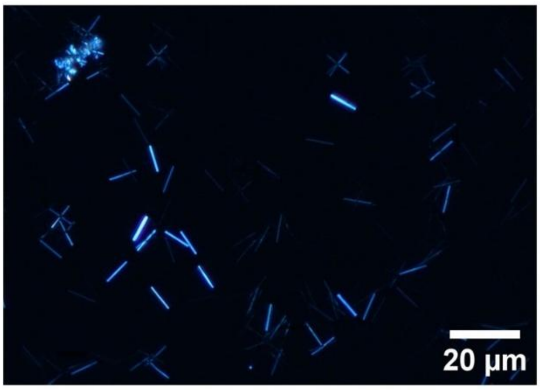

Figure S7: Cross polarized optical microscopy. Birefringence of rod shaped microcrystals formed upon bundling of nanotubes.

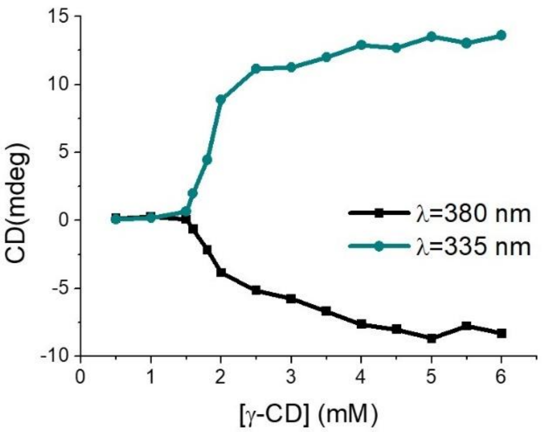

Figure S8: Characterization of inclusion complexes. Formation of the Cotton bands during the titration of the G with increasing amounts of γ-CD ([G]=50μM).

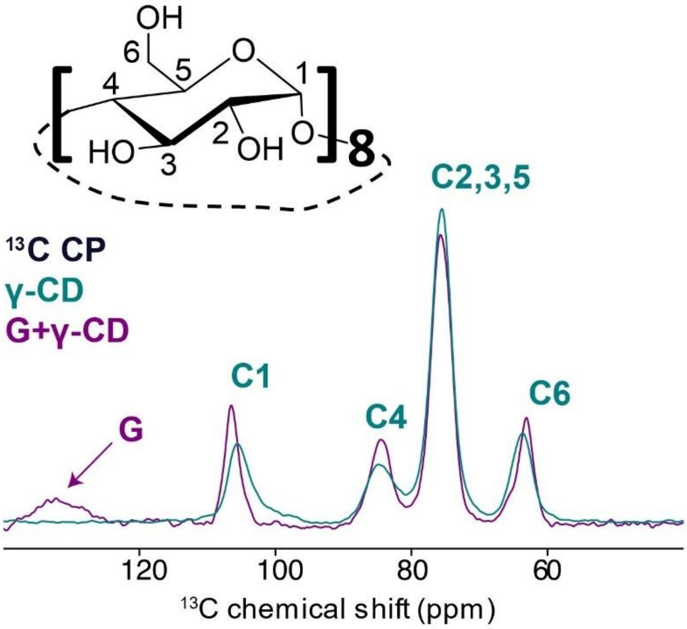

Figure S9: Solid-state NMR. Comparison of the ^{13}C 1D CP-MAS spectrum of lyophilized γ-CD (teal) with the one of the rod-shaped microcrystals formed upon addition of G (purple). Numbered peaks refer to the carbons of the γ-CD, as illustrated in the top of the figure. The broad peak marked with G is attributed to the guest molecule's aromatic carbons.

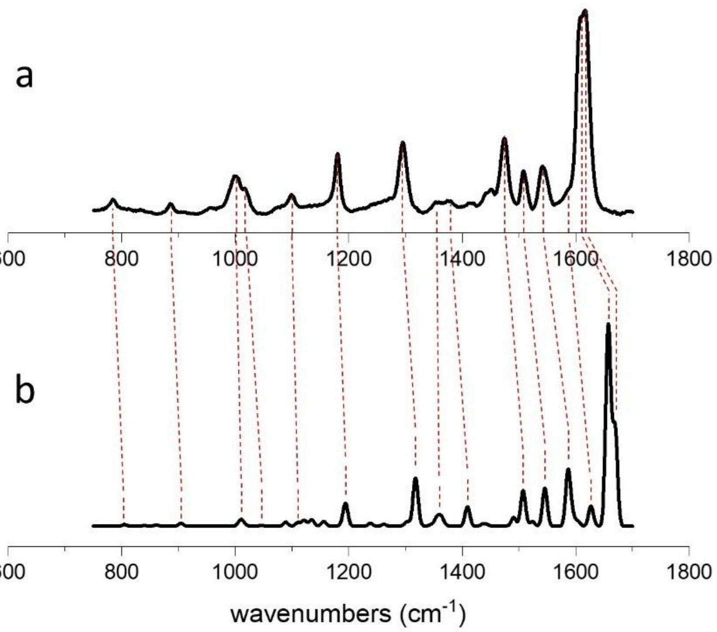

c

Mode	Simulated	Expertimental
	Raman shift (cm^{-1})	
δ(O-H)	1669	1617
ν (C-C)	1657	1607
ν(C-N)	1111	1100
$\delta(C\text{-}H)_{oop}$	1033	1018
ν(C-N)	1007	1005

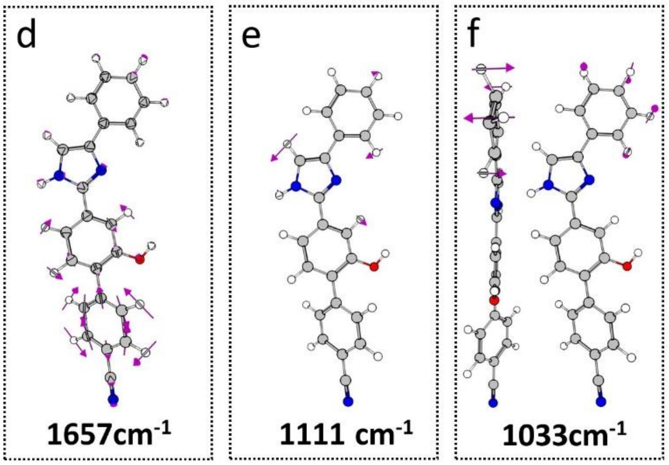

Figure S10: Raman spectroscopy. (**a**)(**b**) Raman vibrational spectra of **G** from (**a**) experimental data and (**b**) DFT calculations. (**c**)(**d**)(**e**)(**f**) Simulated and experimental vibrational modes with respective assignments

Quantum Yield of Emission: The enhancement of photoluminescence upon addition of γ-CD depends on the extent of supramolecular polymerization, which increases at higher [G] and [γ-CD]. For quantum yield measurements of nanotubes, a concentration of [G]=10μM and [γ-CD]=5mM was selected as a compromise to ensure sufficient polymer formation while maintaining low absorbance and minimizing scattering and inner filter effects that could affect measurement accuracy at higher concentrations.

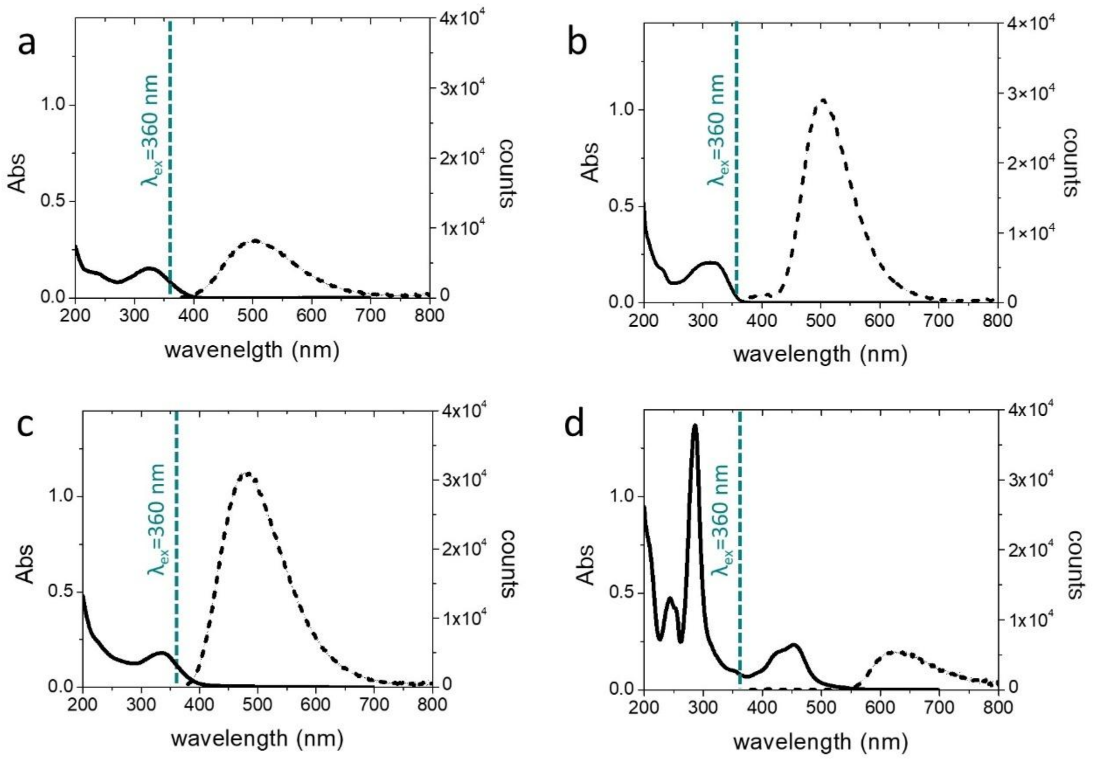

Figure S11: Quantum yield. (**a**)(**b**)(**c**)(**d**) Absorption and emission spectra for used for quantum yield determination (**a**) [G]=10μM (pH=7); (**b**) [G]=10μM (pH=2); (**c**) [G]=10μM in the presence of [γCD]=5mM (pH=7); (**d**) $[Ru(bpy)_3](PF_6)_2$

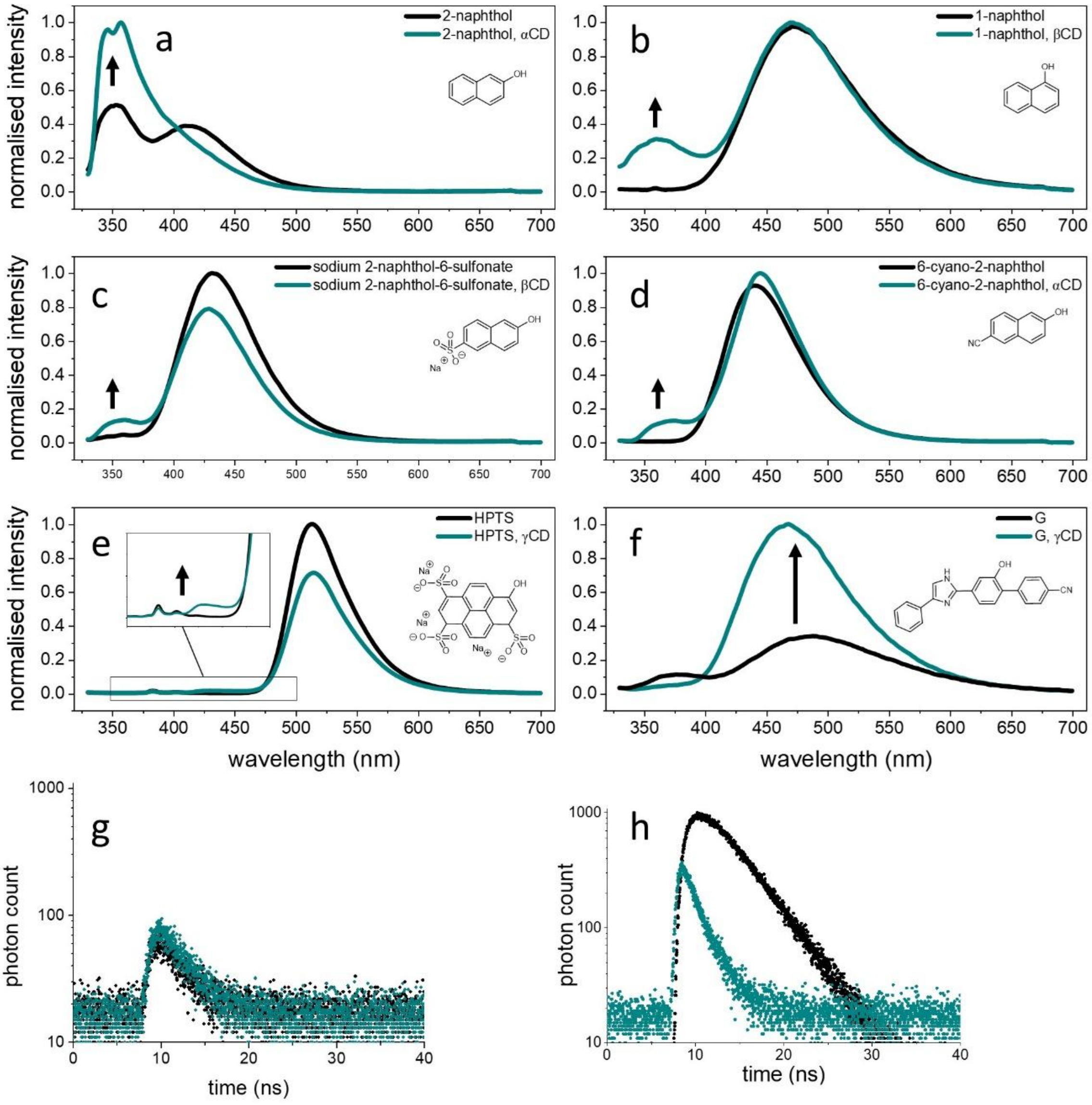

Figure S12: Effects of compartmentalization and lifetime of emission. (**a**)(**b**)(**c**)(**d**)(**e**) Changes in the emission spectra of reported Bronsted photoacids upon addition of cyclodextrins. (**f**) Changes in the emission spectra of G upon addition of γ-cyclodextrins. (**g**) Isotope effect on H-aggregates emission. Fluorescence decay of the H-band recorded by TCSPC in H_2O (black) and D_2O (teal). (**h**) Fluorescence decay from Rhodamine 6G (black), and IRF (teal) recorded by TCSPC.

3. Synthesis

Synthesis of 4'-formyl-2'-hydroxy-[1,1'-biphenyl]-4-carbonitrile

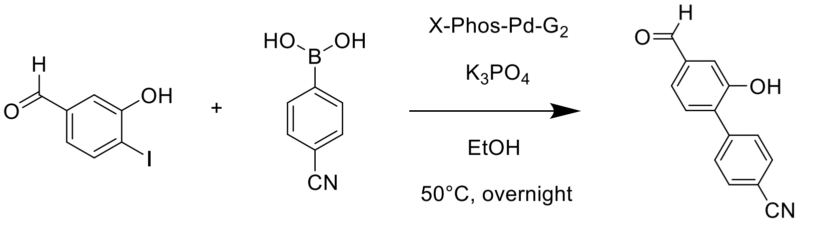

(R1) **(R2)** **1**

A solution of 3-hydroxy-4-iodobenzaldehyde (**R**$_1$) (500 mg, 2.02 mmol) in ethanol (6 mL) was added to a stirred solution of 4-cyanophenyl boronic acid (**R**$_2$)(350 mg, 2.38 mmol), X-Phos-Pd-G2 (64 mg) and potassium phosphate (880 mg) in ethanol (7 mL) under a nitrogen atmosphere. The reaction mixture was degassed by three freeze-pump-thaw cycles, and stirred at 50°C for 16 hours. The crude product was eluted with ethyl acetate over a pad of celite, dried over magnesium sulphate and the solvent was removed under reduced pressure. The crude was purified by column chromatography (silica, MeOH/DCM 1%) to afford **1** with 83 % yield in the form of a white powder.

^{1}H NMR (400 MHz, CD_3OD) δ 9.89 (s, 1H), 7.79 – 7.67 (m, 4H), 7.48 – 7.37 (m, 3H)

^{13}C NMR (101 MHz, CD_3OD) δ 193.6, 156.4, 143.9, 139.0, 134.0, 132.9, 132.3, 131.3, 123.1, 119.8, 116.4, 111.9.

HRMS-ESI Orbitrap (m/z): [M-H$^+$] calculated for $C_{14}H_8NO_2$, 222.0561; found 222.0560

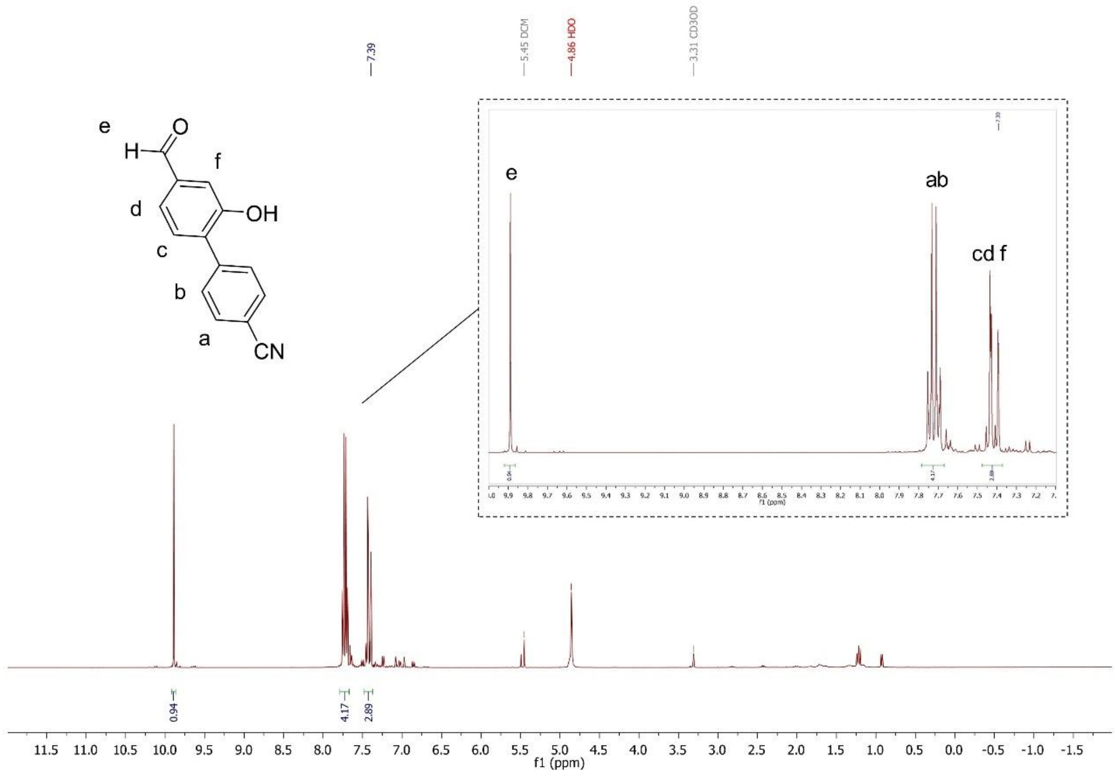

Figure S13: 1H NMR (400 MHz, CD_3OD*) spectrum of 4'-formyl-2'-hydroxy-[1,1'-biphenyl]-4-carbonitrile.*

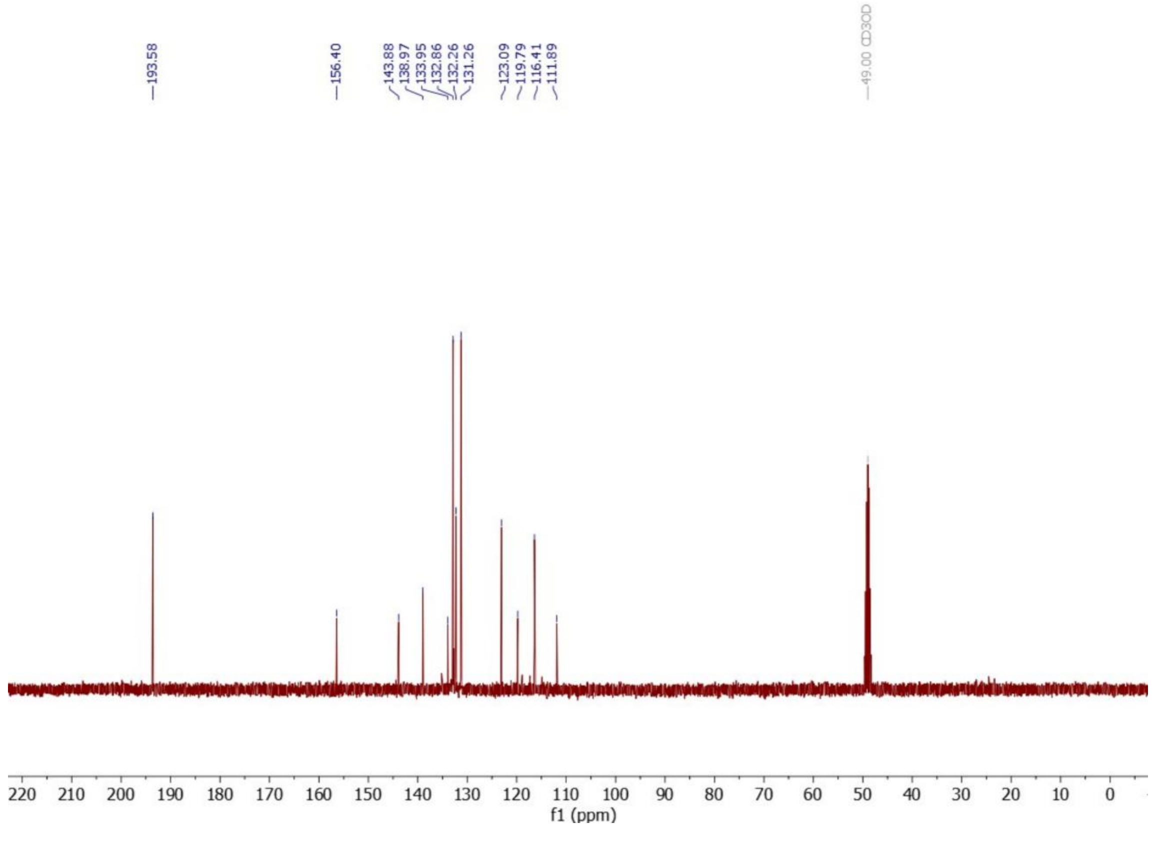

Figure S14: 13C NMR (101 MHz, CD_3OD*) spectrum of 4'-formyl-2'-hydroxy-[1,1'-biphenyl]-4-carbonitrile.*

Synthesis of G

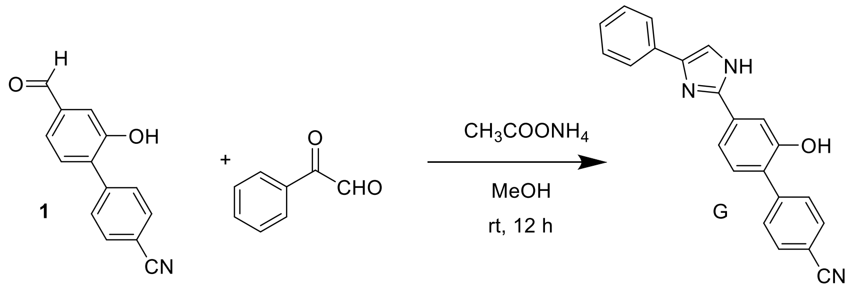

A solution of **1** (350 mg, 1.57 mmol) and ammonium acetate (590 mg, 7.65 mmol) in methanol (10 mL) was added, over a period of 10 minutes, to a solution of phenylglyoxal monohydrate (250 mg, 1.64 mmol) in methanol (12 mL). The reaction mixtures were stirred overnight at room temperature, after which the crude mixture was evaporated under reduced pressure, re-dissolved ethyl acetate and washed with 3 x 50 mL water. The combined organic layers were dried over magnesium sulphate and the solvent was evaporated under reduced pressure. The crude product was purified by column chromatography (silica, MeOH/DCM 1%→MeOH/DCM 3%) to afford the final product (**G**) with 29 % yield as a yellow powder.

Characterization of G

^{1}H NMR (400 MHz, CD_3OD) δ 7.82 – 7.74 (m, 4H), 7.74 – 7.65 (m, 2H), 7.52 (s, 1H), 7.49 – 7.42 (m, 2H), 7.42 – 7.35 (m, 3H), 7.30 – 7.22 (m, 1H).

^{13}C NMR (101 MHz, CD_3OD) δ 156.2, 148.3, 144.7, 132.8, 132.8, 132.0, 131.2, 129.8, 128.3, 128.1, 126.1, 120.0, 118.3, 114.4, 111.1.

HRMS-ESI Orbitrap (m/z): [M+H$^+$] calculated for $C_{22}H_{15}N_3O$, 338.12879; found 338.12860

UV-VIS: λ_{max} (MeOH) = 331 nm ($\log_{10}$ ε 4.5)

FTIR: ν (C-C) 1599 cm^{-1} (sh, m); ν (C≡N) 2222 cm^{-1}(sh, m)

Raman spectrum: ν (C-C) 1607 cm^{-1} (sh, s); ν (C≡N) 2223 cm^{-1}(sh, w)

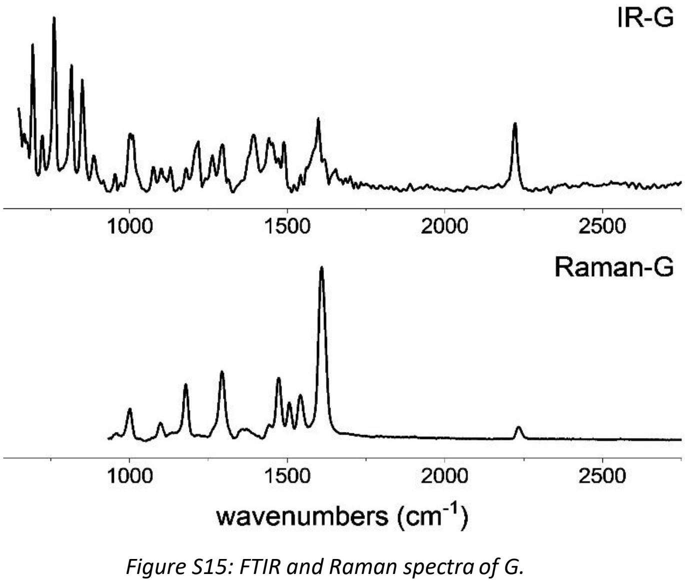

Figure S15: FTIR and Raman spectra of G.

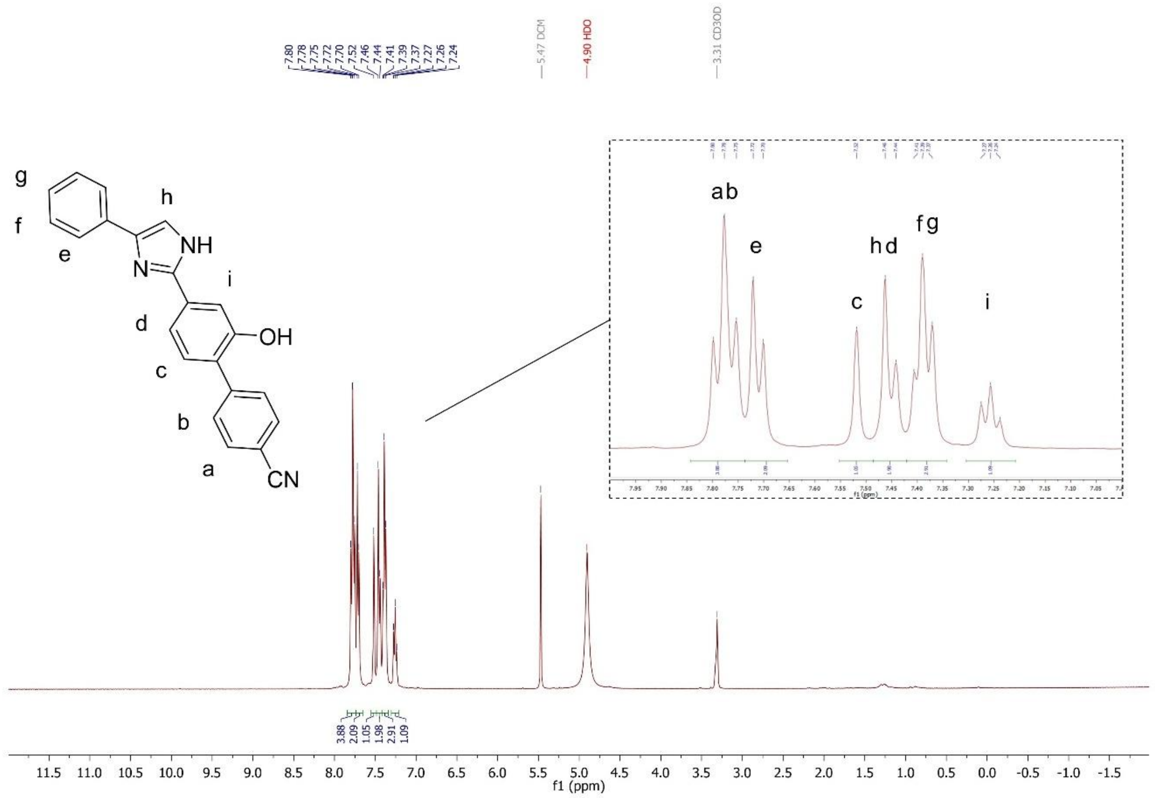

Figure S16: ^{1}H NMR (400 MHz, CD_3OD) spectrum of G.

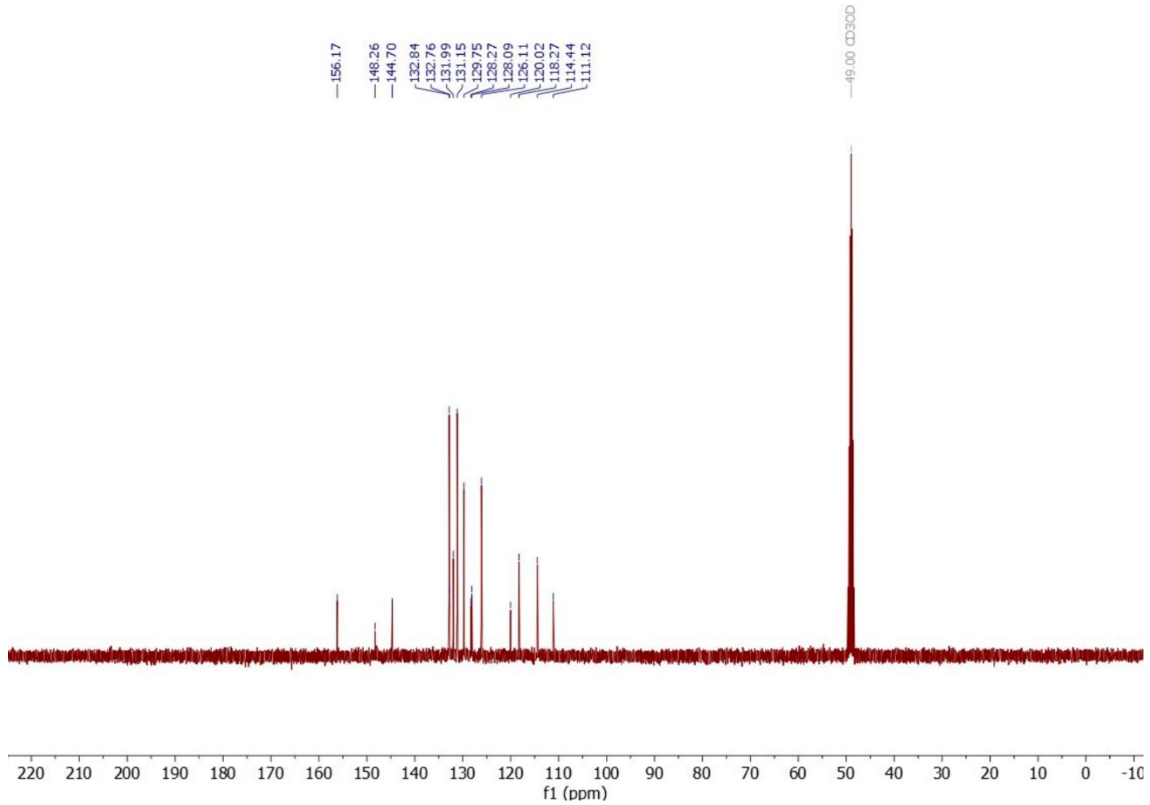

Figure S17: ^{13}C NMR (101 MHz, CD_3OD) spectrum of G.

4. Computational methods

Initial molecular geometries were constructed using the Chemcraft software. Quantum chemical calculations were performed using density functional theory (DFT) as implemented in the ORCA software.[12] Geometry optimizations were carried out at the B3LYP-D3(BJ)/def2-SVP level of theory.[13-18] The D3 dispersion correction with Becke–Johnson damping was applied to account for dispersion interactions.[19-20] The RIJCOSX approximation, in conjunction with the def2-J auxiliary basis set, was used to accelerate integral evaluation.[21-22] Solvation effects from water were accounted for using the CPCM model.[23] All calculations were performed without symmetry constraints.

Vibrational frequency calculations were performed at the same level of theory using numerical differentiation (NUMFREQ) to confirm that the optimized structures correspond to true minima (no imaginary frequencies). Raman activities were obtained from finite-difference derivatives of the polarizability tensor. Simulated Raman spectra were generated by applying Gaussian broadening (10 cm^{-1} FWHM) to the calculated Raman activities using the ORCA utility *orca_mapspc* over the 0–14000 cm^{-1} range.

Time-dependent DFT (TD-DFT) calculations were carried out on the optimized geometries using the CAM-B3LYP functional in combination with the def2-SVP basis set to account for long-range corrected excitations.[24] Transition dipole moments were extracted from these calculations.

The molecular volume of G was estimated computationally by first optimizing the structure using the semi-empirical GFN2-xTB method from Grimme and co-workers (as implemented in its standalone code),[25] and then using Monte Carlo method requested with the *Volume* keyword in Gaussian 16.[26] The calculated volume was 394.93 $Å^3$/mol.

Cartesian coordinates of computed geometry for the guest:

C	3.25596546092706	2.08533224130959	-0.70619868949717
C	3.65047406846708	3.39232838359734	-0.35380048823962
C	4.97789019284276	3.79702978080677	-0.44548349548143
C	5.95459252470282	2.89101101028692	-0.89923365381426
C	5.57792579134098	1.58219110433670	-1.25639059528208
C	4.24774598744431	1.18957487333891	-1.15640240122987
C	1.82775105579524	1.69874256586391	-0.63172924898950
C	1.40529088264345	0.42563396461494	-0.17165002242613
C	0.04540632303584	0.11339718726549	-0.10458027044597
C	-0.93623424578056	1.03958835870004	-0.48937284552978
C	-0.52791739828831	2.30494603100480	-0.94816552976340
C	0.82774823361141	2.61432321909263	-1.00655314812436
O	2.34592609137079	-0.46584282955875	0.22214840017909
C	-2.34473471233339	0.65048658503384	-0.39837091632014
N	-3.39836269682541	1.47729663534607	-0.68746485102181
C	-4.55674839361223	0.77746909289538	-0.48770982386323
C	-4.16469216124362	-0.49004524628817	-0.07142887667205
N	-2.79041635007928	-0.54198439219729	-0.02474558560261
C	-5.00666875207409	-1.64259385910843	0.27882304882544

C	-4.41493607493032	-2.86098098306314	0.66144576303299
C	-5.20302427421179	-3.96422106002384	0.99536669375457
C	-6.59861814511766	-3.87410238604502	0.95489915428875
C	-7.19829952757817	-2.66679600974927	0.57656443099633
C	-6.41249251557344	-1.56285129065858	0.24224931091802
C	7.32471151950975	3.29766237720203	-0.99536050208274
N	8.43646302689462	3.62746978775120	-1.07383008882195
H	2.90582096060502	4.09930395496687	0.01587805453965
H	5.26540881881145	4.81059065564060	-0.16043836575948
H	6.33204168726762	0.87955161093809	-1.61539923291805
H	3.96912687913683	0.17570969016736	-1.43957733630449
H	-0.26217589174976	-0.86837547515872	0.26118365611324
H	-1.25509283249483	3.05173129026205	-1.27040389017779
H	1.12901055015604	3.59523022996697	-1.37922496063032
H	1.92035102604259	-1.28026007588556	0.53229442926828
H	-3.34839445294908	2.44493540637817	-0.98887562908207
H	-5.53312392136398	1.22421377614605	-0.65322462823677
H	-3.32645821584780	-2.92862887916253	0.69206262792007
H	-4.72364058697011	-4.90159954194017	1.28945350905662
H	-7.21530278417448	-4.73748820357987	1.21623968551924
H	-8.28760968658837	-2.58406080766449	0.54193116374126
H	-6.89790746081896	-0.62881877282893	-0.04962485183649

5. References

[1] A. Sorrenti, J. Leira-Iglesias, A. J. Markvoort, T. F. A. de Greef, T. M. Hermans, "Non-Equilibrium Supramolecular Polymerization," *Chemical Society Reviews* 46, no. 18 (2017): 5476–5490. https://doi.org/10.1039/c7cs00121e.

[2] A. T. R. Williams, S. A. Winfield, J. N. Miller, "Relative Fluorescence Quantum Yields Using a Computer-Controlled Luminescence Spectrometer," *Analyst* 108, no. 1290 (1983): 1067–1071. https://doi.org/10.1039/an9830801067.

[3] L. Stryer, "Excited-State Proton-Transfer Reactions. A Deuterium Isotope Effect on Fluorescence," *Journal of the American Chemical Society* 88, no. 24 (1966): 5708–5712. https://doi.org/10.1021/ja00976a004.

[4] A. Altomare, C. Cuocci, C. Giacovazzo, A. Moliterni, R. Rizzi, N. Corriero, et al., "EXPO2013: A Kit of Tools for Phasing Crystal Structures from Powder Data," *Journal of Applied Crystallography* 46, no. 4 (2013): 1231–1235. https://doi.org/10.1107/S0021889813013113.

[5] A. E. Bennett, C. M. Rienstra, M. Auger, K. V. Lakshmi, R. G. Griffin, "Heteronuclear Decoupling in Rotating Solids," *Journal of Chemical Physics* 103, no. 16 (1995): 6951–6958. https://doi.org/10.1063/1.470372.

[6] F. Delaglio, S. Grzesiek, G. W. Vuister, G. Zhu, J. Pfeifer, A. Bax, "NMRPipe: A Multidimensional Spectral Processing System Based on UNIX Pipes," *Journal of Biomolecular NMR* 6, no. 3 (1995): 277–293. https://doi.org/10.1007/BF00197809.

[7] T. J. Stevens, R. H. Fogh, W. Boucher, V. A. Higman, F. Eisenmenger, B. Bardiaux, et al., "A Software Framework for Analysing Solid-State MAS NMR Data," *Journal of Biomolecular NMR* 51, no. 4 (2011): 437–447. https://doi.org/10.1007/s10858-011-9569-2.

[8] C. R. Morcombe, K. W. Zilm, "Chemical Shift Referencing in MAS Solid State NMR," *Journal of Magnetic Resonance* 162, no. 2 (2003): 479–486. https://doi.org/10.1016/s1090-7807(03)00082-x.

[9] O. H. Kwon, O. F. Mohammed, "Water-Wire Catalysis in Photoinduced Acid-Base Reactions," *Physical Chemistry Chemical Physics* 14, no. 25 (2012): 8974–8980. https://doi.org/10.1039/c2cp23796b.

[10] O. H. Kwon, Y. S. Lee, B. K. Yoo, D. J. Jang, "Excited-State Triple Proton Transfer of 7-Hydroxyquinoline Along a Hydrogen-Bonded Alcohol Chain: Vibrationally Assisted Proton Tunneling," *Angewandte Chemie International Edition* 45, no. 3 (2006): 415–419. https://doi.org/10.1002/anie.200503209.

[11] K. I. Assaf, W. M. Nau, "The Chaotropic Effect as an Assembly Motif in Chemistry," *Angewandte Chemie International Edition* 57, no. 43 (2018): 13968–13981. https://doi.org/10.1002/anie.201804597.

[12] F. Neese, "The ORCA Program System," *WIREs Computational Molecular Science* 2, no. 1 (2012): 73–78. https://doi.org/10.1002/wcms.81.

[13] A. D. Becke, "Density-Functional Thermochemistry. III. The Role of Exact Exchange," *Journal of Chemical Physics* 98, no. 7 (1993): 5648–5652. https://doi.org/10.1063/1.464913.

[14] C. Lee, W. Yang, R. G. Parr, "Development of the Colle-Salvetti Correlation-Energy Formula into a Functional of the Electron Density," *Physical Review B* 37, no. 2 (1988): 785–789. https://doi.org/10.1103/physrevb.37.785.

[15] S. H. Vosko, L. Wilk, M. Nusair, "Accurate Spin-Dependent Electron Liquid Correlation Energies for Local Spin-Density Calculations: A Critical Analysis," *Canadian Journal of Physics* 58, no. 8 (1980): 1200–1211. https://doi.org/10.1139/p80-159.

[16] P. J. Stephens, F. J. Devlin, C. F. Chabalowski, M. J. Frisch, "Ab Initio Calculation of Vibrational Absorption and Circular-Dichroism Spectra Using Density-Functional Force-Fields," *Journal of Physical Chemistry* 98, no. 45 (1994): 11623–11627. https://doi.org/10.1021/j100096a001.

[17] F. Weigend, R. Ahlrichs, "Balanced Basis Sets of Split Valence, Triple Zeta Valence and Quadruple Zeta Valence Quality for H to Rn: Design and Assessment of Accuracy," *Physical Chemistry Chemical Physics* 7, no. 18 (2005): 3297–3305. https://doi.org/10.1039/b508541a.

[18] C. Alkemade, H. Wierenga, V. A. Volkov, M. Preciado López, A. Akhmanova, P. R. Ten Wolde, et al., "Cross-Linkers at Growing Microtubule Ends Generate Forces that Drive Actin Transport," *Proceedings of the National Academy of Sciences of the United States of America* 119, no. 11 (2022). https://doi.org/10.1073/pnas.2112799119.

[19] S. Grimme, S. Ehrlich, L. Goerigk, "Effect of the Damping Function in Dispersion Corrected Density Functional Theory," *Journal of Computational Chemistry* 32, no. 7 (2011): 1456–1465. https://doi.org/10.1002/jcc.21759.

[20] S. Grimme, J. Antony, S. Ehrlich, H. Krieg, "A Consistent and Accurate Ab Initio Parametrization of Density Functional Dispersion Correction (DFT-D) for the 94 Elements H-Pu," *Journal of Chemical Physics* 132, no. 15 (2010). https://doi.org/10.1063/1.3382344.

[21] F. Neese, F. Wennmohs, A. Hansen, U. Becker, "Efficient, Approximate and Parallel Hartree–Fock and Hybrid DFT Calculations. A 'Chain-of-Spheres' Algorithm for the Hartree–Fock Exchange," *Chemical Physics* 356, no. 1–3 (2009): 98–109. https://doi.org/10.1016/j.chemphys.2008.10.036.

[22] F. Weigend, "Accurate Coulomb-Fitting Basis Sets for H to Rn," *Physical Chemistry Chemical Physics* 8, no. 9 (2006): 1057–1065. https://doi.org/10.1039/B515623H.

[23] V. Barone, M. Cossi, "Quantum Calculation of Molecular Energies and Energy Gradients in Solution by a Conductor Solvent Model," *Journal of Physical Chemistry A* 102, no. 11 (1998): 1995–2001. https://doi.org/10.1021/jp9716997.

[24] T. Yanai, D. P. Tew, N. C. Handy, "A New Hybrid Exchange–Correlation Functional Using the Coulomb-Attenuating Method (CAM-B3LYP)," *Chemical Physics Letters* 393, no. 1–3 (2004): 51–57. https://doi.org/10.1016/j.cplett.2004.06.011.

[25] C. Bannwarth, S. Ehlert, S. Grimme, "GFN2-xTB—An Accurate and Broadly Parametrized Self-Consistent Tight-Binding Quantum Chemical Method with Multipole Electrostatics and Density-Dependent Dispersion Contributions," *Journal of Chemical Theory and Computation* 15, no. 3 (2019): 1652–1671. https://doi.org/10.1021/acs.jctc.8b01176.

[26] M. J. Frisch, G. W. Trucks, H. B. Schlegel, G. E. Scuseria, M. A. Robb, J. R. Cheeseman, et al., Gaussian 16, Revision C.01, Gaussian, Inc., Wallingford, CT, 2016.
